# Spatiotemporal mapping of malaria incidence in Sudan using routine surveillance data

**DOI:** 10.1038/s41598-022-16706-1

**Published:** 2022-08-18

**Authors:** Ahmed Elagali, Ayman Ahmed, Nada Makki, Hassan Ismail, Mark Ajak, Kefyalew Addis Alene, Daniel J. Weiss, Abdalla Ahmed Mohammed, Mustafa Abubakr, Ewan Cameron, Peter Gething, Asmaa Elagali

**Affiliations:** 1 Telethon Kids Institute, Perth, WA Australia; 2 Department of Zoology, Omdurman Islamic University, Khartoum, Sudan; 3 Institute of Endemic Diseases, University of Khartoum, Khartoum, 11111 Sudan; 4 Swiss Tropical and Public Health Institute (Swiss TPH), 4123 Allschwil, Switzerland; 5 University of Basel, Petersplatz 1, 4001 Basel, Switzerland; 6 Health Information Management and Statistics, Federal Ministry of Health, Khartoum, Sudan; 7 Neglected Tropical Diseases Control Division, Federal Ministry of Health, Khartoum, Sudan; 8 Preventive Health Services, National Ministry of Health, Juba, South Sudan; 9 Faculty of Health Sciences, Curtin University, Perth, WA Australia; 10 Diseases Control Directorate, Federal Ministry of Health, Khartoum, Sudan; 11 Department of the Integrated Vector Management (IVM), Federal Ministry of Health, Khartoum, Sudan

**Keywords:** Medical research, Epidemiology

## Abstract

Malaria is a serious threat to global health, with over $95\%$ of the cases reported in 2020 by the World Health Organization in African countries, including Sudan. Sudan is a low-income country with a limited healthcare system and a substantial burden of malaria. The epidemiology of malaria in Sudan is rapidly changing due to factors including the rapidly developing resistance to drugs and insecticides among the parasites and vectors, respectively; the growing population living in humanitarian settings due to political instability; and the recent emergence of *Anopheles stephensi* in the country. These factors contribute to changes in the distribution of the parasites species as well as malaria vectors in Sudan, and the shifting patterns of malaria epidemiology underscore the need for investment in improved situational awareness, early preparedness, and a national prevention and control strategy that is updated, evidence based, and proactive. A key component of this strategy is accurate, high-resolution endemicity maps of species-specific malaria. Here, we present a spatiotemporal Bayesian model, developed in collaboration with the Sudanese Ministry of Health, that predicts a fine-scale (1 km $\times$ 1 km) clinical incidence and seasonality profiles for *Plasmodium falciparum* and *Plasmodium vivax* across the country. We use monthly malaria case counts for both species collected via routine surveillance between January 2017 and December 2019, as well as a suite of high-resolution environmental covariates to inform our predictions. These epidemiological maps provide a useful resource for strategic planning and cost-effective implementation of malaria interventions, thus informing policymakers in Sudan to achieve success in malaria control and elimination.

## Introduction

Mosquito-borne diseases yield a huge burden of disease around the world and lead to about 700 million in cases and 1 million in deaths from mosquito-borne diseases each year^1–3^. Malaria is a mosquito-borne protozoan disease caused by one of the five species of Plasmodium parasites, namely *Plasmodium falciparum*, *P. ovale*, *P. malariae*, *P. vivax* and *P. knowlesi*^4^. Malaria is currently endemic in tropical and subtropical areas in Africa, Asia as well as South America. According to the World Health Organisation (WHO) estimates, in 2020 there were 241 million in malaria cases in the 85 malaria-endemic countries, leading to 627, 000 deaths. Of these cases, $96\%$ occurred in Africa^4^. Estimated malaria-related morbidity and mortality have increased by $6\%$ and $12\%$, respectively, from 2019 to 2020, partly due to disruptions to malaria prevention and treatment services caused by the COVID-19 pandemic^4–6^.

From 2000 to 2020, malaria incidence in the WHO Eastern Mediterranean Region (EMRO) decreased from 21 to 11 cases (per 1000 populations at risk). This decline, coupled with population growth, led to a drop in cases from 7 million to 5.7 million cases^4^. The majority of this decline was driven by intervention efforts including increased availability of artemisinin-based combination therapy (ACT), indoor residual spraying (IRS), and insecticide-treated bed nets (ITNs)^7^. The Republic of Sudan has the highest malaria incidence in the EMRO Region, contributing $56\%$ of estimated cases^4^. Despite the declining malaria incidence rates within the Region, cases in Sudan nearly doubled between 2015 and 2019^4,6,8^. This alarming increase is likely the result of many intertwined factors. In the past 3 years (2018–2020) Sudan had experienced unusually heavy rainy seasons that caused flash floods^9^. In 2018, these floods caused difficult operational environments leading to humanitarian emergencies as well as a huge population displacements, with over 200, 000 people in 15 states affected/displaced. The overall economic difficulties that the country is facing, as well as ongoing political instability, conflicts, and the growing size of the population living in humanitarian settings in the country, contribute to the complex epidemiological situation in Sudan. These factors are potentially as important to malaria transmission as climate change and environmental drivers such as temperature and precipitation^9,10^.

In Sudan, the transmission of malaria is believed to have been sustained almost entirely by *Anopheles arabiensis* along the Nile Valley north of Khartoum to the southern Egyptian border^11,12^. Other malaria vectors in Sudan include *An. gambiae s.s.*, *An. pharoensis* and *An. funestus* but these are rare and limited only to the eastern and southern part of the country^13,14^. In the year 2019, the presence of the invasive Asian malaria vector *An. stephens* was reported in Sudan for the first time, which increased malaria risk in urban areas^15–18^. This vector is rapidly spreading and expanding it is geographical distribution in the Horn of Africa^19,20^. Unlike native malaria vectors in Africa, *An. stephensi* breeds mainly in water containers and quickly adapt to harsh local environments^21^. It can tolerate the dry season’s high temperatures, when malaria transmission is at its lowest levels, thus increasing potential transmission during the dry seasons. Furthermore, resistance to the four classes of IRS and ITNs recommended by the WHO has been reported in several populations of *An. stephensi’s*, increasing the challenge for the vector control^6,18,22^.

**Figure 1:**
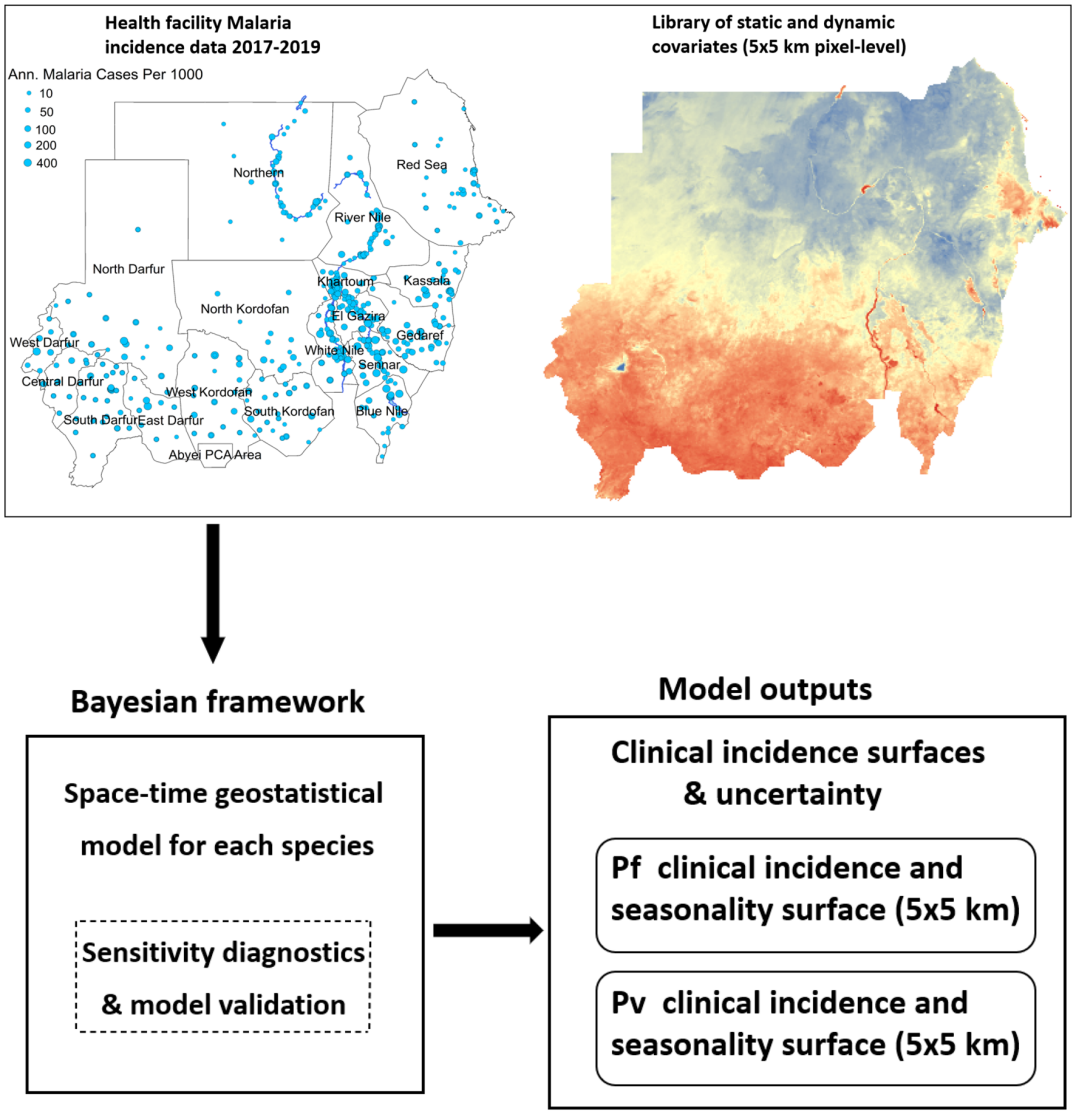
An overview of the input data, Bayesian framework structure and model outputs. This figure was created in R (https://www.r-project.org/) using the ggplot package.

The landscape in Sudan extends over five ecological zones, including the Sahara in the North, coastal and sub-coastal areas in the east, poor Savannah in the central and western regions, the rich Savannah and mountain vegetation regions in the South. As a result, there is a noticeable gradient in the endemicity of malaria across the country^23^. Malaria is considered hypoendemic in the desert fringe in the Northern, Red Sea (excluding Port Sudan), River Nile and North Darfour (excluding Elfashir) states. The Eastern, Southern and Western parts of Sudan fluctuate between mesoendemic and hyperendemic depending on seasonal rainfall and the associated mosquitoes’ abundance. These naturally occurring environmental conditions influence malaria epidemiology along with areas impacted by large-scale irrigation projects, conflicts, and humanitarian crises. Irrigated areas constitute around $1.5\%$ of the total farmland, in Al-Gezira, Elzidab, Asalia, Kinana, New Halafa, West Sinnar and Elrahad, thereby increasing suitable mosquito breeding sites and stable transmission of malaria throughout the year. The emergency and conflict regions are mainly due to internally displaced populations (IDP) from conflicts in Darfour, Kordofan and Abyei, as well as from neighbouring countries. In 2018, the number of vulnerable refugees and asylum seekers reached 1.2 million, whereas the total number of IDPs mounted to 3 million.

The two main dominant malaria species in Sudan are *Plasmodium falciparum* ($\sim 87.6\%$), and *Plasmodium vivax* ($\sim 8.1\%$), with *Plasmodium ovale* and coinfections of both *Plasmodium falciparum* and *Plasmodium vivax* representing the remaining infections in the country (see table 1, Sudan MIS 2016). Generally, the *Plasmodium vivax* prevalence varies between states and is higher in IDP camps compared to rural and urban areas. It is also higher in children, males, pregnant women and lower wealth quintiles compared to adults, females and non-pregnant women (Sudan MIS 2016). *Plasmodium vivax* and mixed infections were found to be higher in urban ($16.9\%$) than in rural areas (*P*-value $= <0.0001$; Sudan MIS 2016). However, the dominant *Plasmodium* species varies among states in Sudan^24–26^. In the past, *Plasmodium falciparum* caused more than $95\%$ of malaria cases in Sudan, but the proportion of *Plasmodium vivax* cases has been increasing throughout the country in recent years. Severe *Plasmodium vivax* malaria is more prevalent in eastern parts of Sudan and the states bordering Ethiopia^25^. A possible cause of the increase in *Plasmodium vivax* malaria was the influx of migrants from Eritrea and Ethiopia following the construction of roads that link and facilitate the travel between these three countries. However, human migrations do not explain the presence of *Plasmodium vivax* in other parts of Sudan, particularly the western states. This suggests that *Plasmodium vivax* is adapting to new epidemiological contexts and challenging the notion of refractory Duffy-negative populations^27^.

In recent years, the transmission of malaria has changed and is more heterogeneous than in the past due to the various malaria control strategies implemented by endemic countries in collaboration with the WHO. Among these strategies is the subnational level risk stratification in accordance with past and present malaria risk. Stratifying malaria risk within a country provides a mechanism for identifying geographically-specific malaria intervention strategies. To bring forward a complete stratification plan, local malaria data and high-resolution burden maps are essential, especially for optimising the mixture of interventions to maximize the impact and cost-efficacy in each stratum. Such strategies are crucial for combating malaria in countries such as Sudan that have very limited resources and weak health systems. In this article, we present the results of a spatiotemporal Bayesian analysis designed to reveal the distinct spatiotemporal patterns of *Plasmodium falciparum* and *Plasmodium vivax* malaria endemicity in Sudan, along with the seasonal profiles of these two diseases (see Fig. 1). This Bayesian framework takes fine-scale resolution environmental covariates and monthly clinical incidence reports from healthcare facilities as inputs. The clinical incidence reports were obtained from the National Malaria Control Program for 3 years (2017–2019). The resulting maps of malaria incidence will provide a valuable resource for the Ministry of Health in Sudan to plan public health interventions and structure cost-effective malaria control strategies. We provide a detailed description of our modelling framework and validation approach in the Materials and methods section of the paper.

**Table 1:** The percentage of malaria parasite prevalence in Sudan according to residential status.

Residence	*Plasmodium falciparum*%	*Plasmodium vivax*%	Co-infections and others%
Urban	80.4	16.9	2.6
Rural	88.2	6.7	5.2
IDPs camps	94.8	3.1	2.1

These data were published in the Sudan MIS 2016 report.

## Results

### *Plasmodium falciparum* fine scale endemicity surface

**Figure 2:**
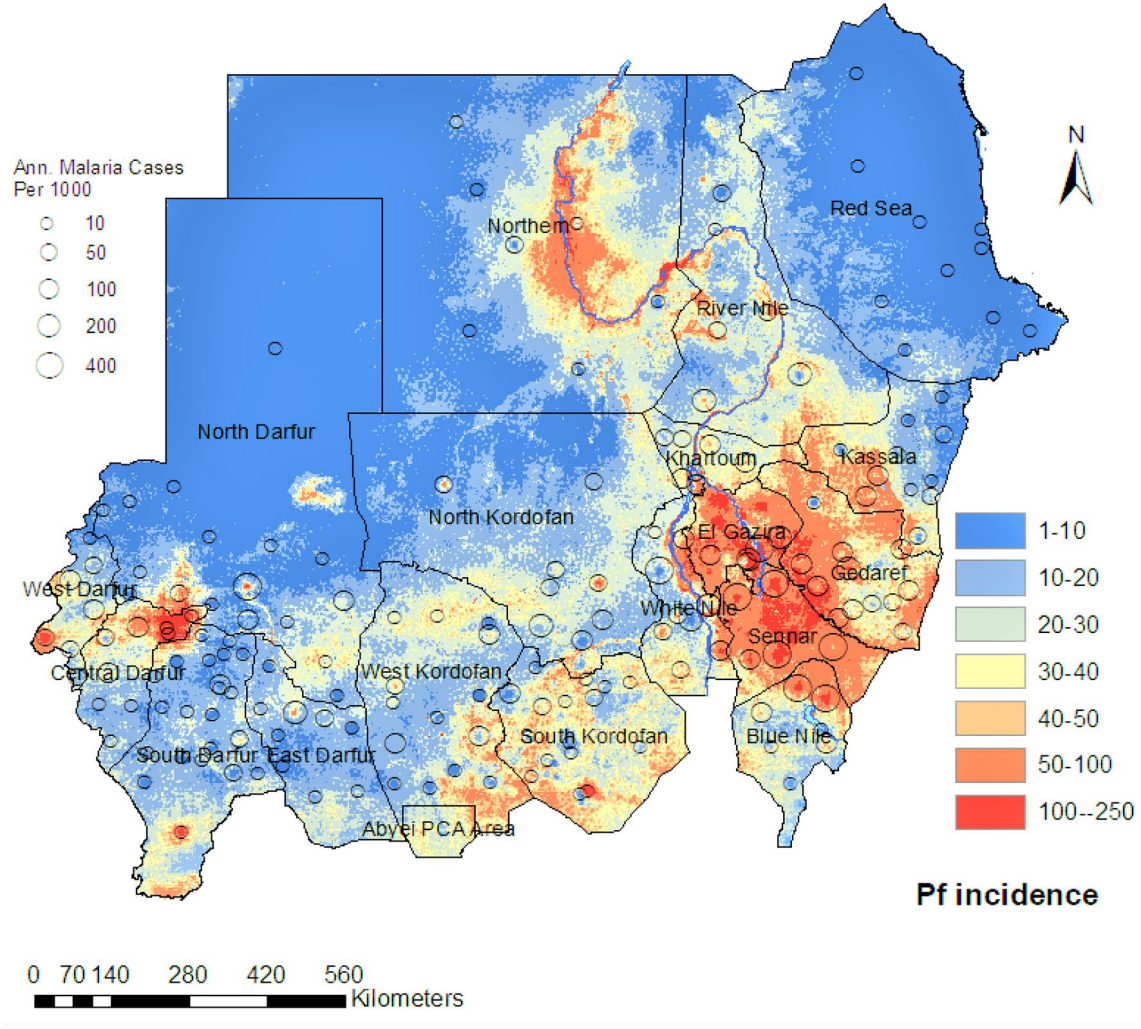
A fine-scale map ($1\times 1\,$km) of the estimated annual incidence of clinical *Plasmodium falciparum* malaria per 1000 in Sudan for years 2017–2019. Hollow circles represent reported cases per year within each locality. Colour shading represents predicted values derived from a Bayesian geostatistical model.

The fine-scale map ($1\times 1\,$km) of *Plasmodium falciparum* malaria incidence in Sudan, which was inferred based on our spatiotemporal Bayesian model and fit to the yearly routine surveillance data between 2017 and 2019, is shown in Fig. 2. This map reveals the spatial heterogeneity of *Plasmodium falciparum* malaria incidence in Sudan. The *Plasmodium falciparum* transmission gradient in Sudan aligns with the ecological regions of the country^10,23^. *Plasmodium falciparum* incidence in the northern part of Sudan is considered hypoendemic, as this region has high levels of aridity except for the parts close to the river Nile. River adjacent areas have high levels of malaria transmission due to the proximity to the water source and the associated mosquito breeding sites. There are several regions in the country with a higher *Plasmodium falciparum* malaria burden overall, including the central region (Khartoum area), the western and south-western region (Darfour and south Kordofan), and the eastern regions (Sennar, White Nile, and Al-Gezira). In South Kordofan region, malaria cases in 2019 equalled $5.5\%$ of the state’s population^10^. In the Darfour area, West Darfour has the highest *Plasmodium falciparum* malaria burden, this sub-region has been at the centre of the ongoing Darfour conflict and thousands of residents have been regularly displaced from their homes.

**Figure 3:**
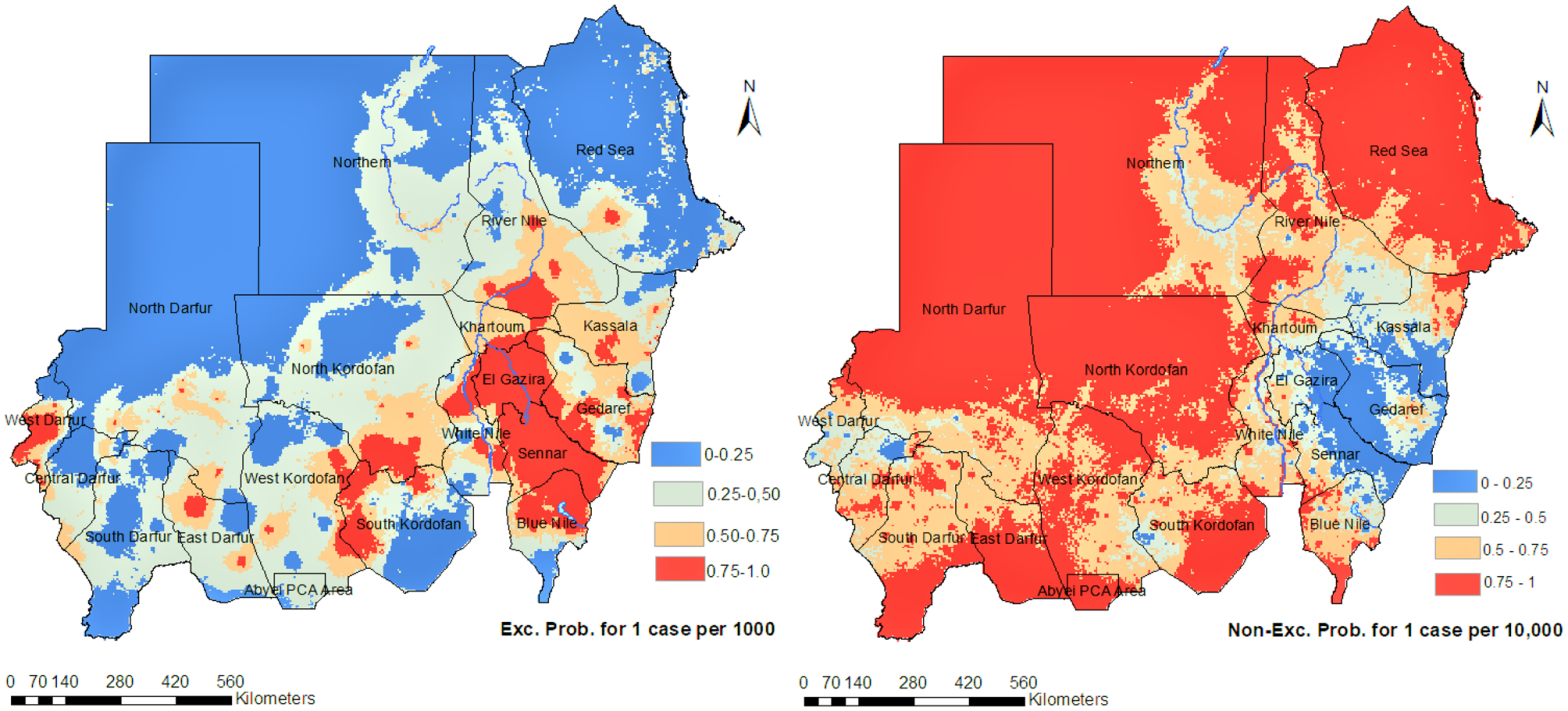
The posterior probability that the incidence cases of the *P. falciparum* malaria in Sudan exceeds 1 case per 1000 Person-Year-Observed (left panel) and does not exceed 1 case per 10,000 Person-Year-Observed (right panel) in each pixel based on our spatiotemporal Bayesian model fit. These maps were created in ArcGIS (https://www.arcgis.com) using the ArcMap package.

**Figure 4:**
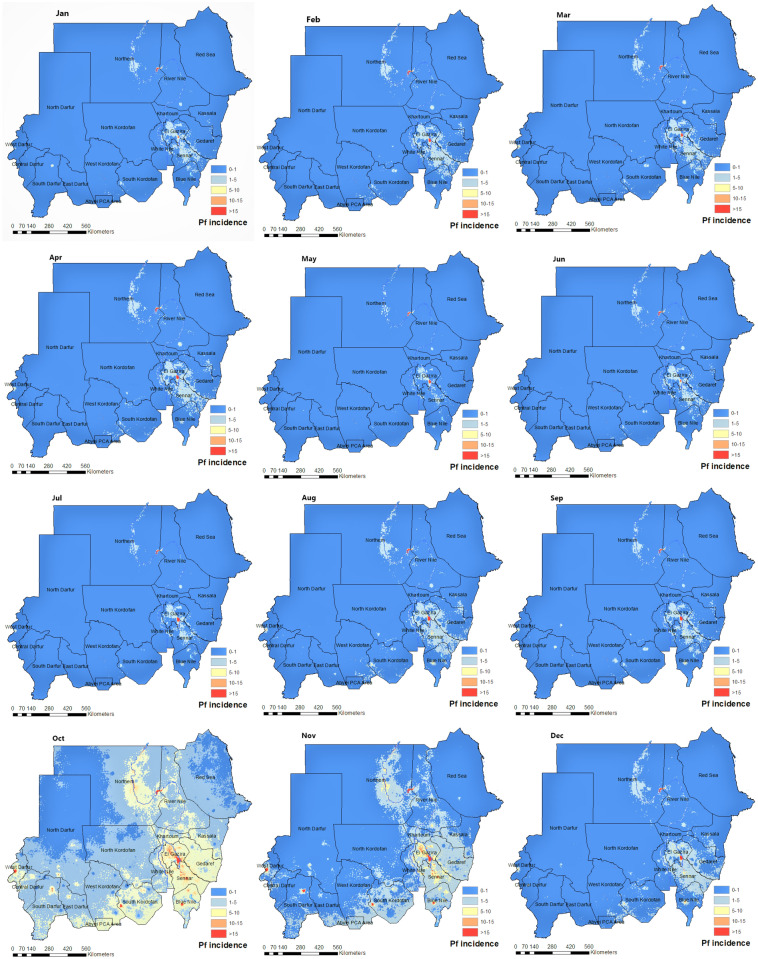
A fine-scale map ($1\times 1\,$km) of the incidence cases of the *P. falciparum* malaria per 1000 in each calendar month in Sudan inferred based on our spatiotemporal Bayesian model fit to the monthly routine surveillance data between 2017 and 2019. These maps were created in ArcGIS (https://www.arcgis.com) using the ArcMap package.

Figure 3 shows the posterior probability from our spatiotemporal Bayesian model fit that the incidence of *Plasmodium falciparum* malaria in Sudan exceeds 1 case per 1000 person-Year-Observed (upper panel) and does not exceed 1 case per 10, 000 (lower panel). These maps provide a resource to identify high transmission areas, with associated uncertainty, and are amenable for use in risk stratification and malaria intervention planning. Moreover, Fig. 4 illustrates a fine-scale map ($1\times 1\,$km) of *Plasmodium falciparum* incidence of the based on our spatiotemporal Bayesian model fit. Maps were made for each calendar month in Sudan based on the monthly routine surveillance data between 2017 and 2019. The surveillance data and modelled results support our understanding that the peak season of malaria transmission occurs between October and December, following the rainy season that typically occurs from June to early November.

**Figure 5:**
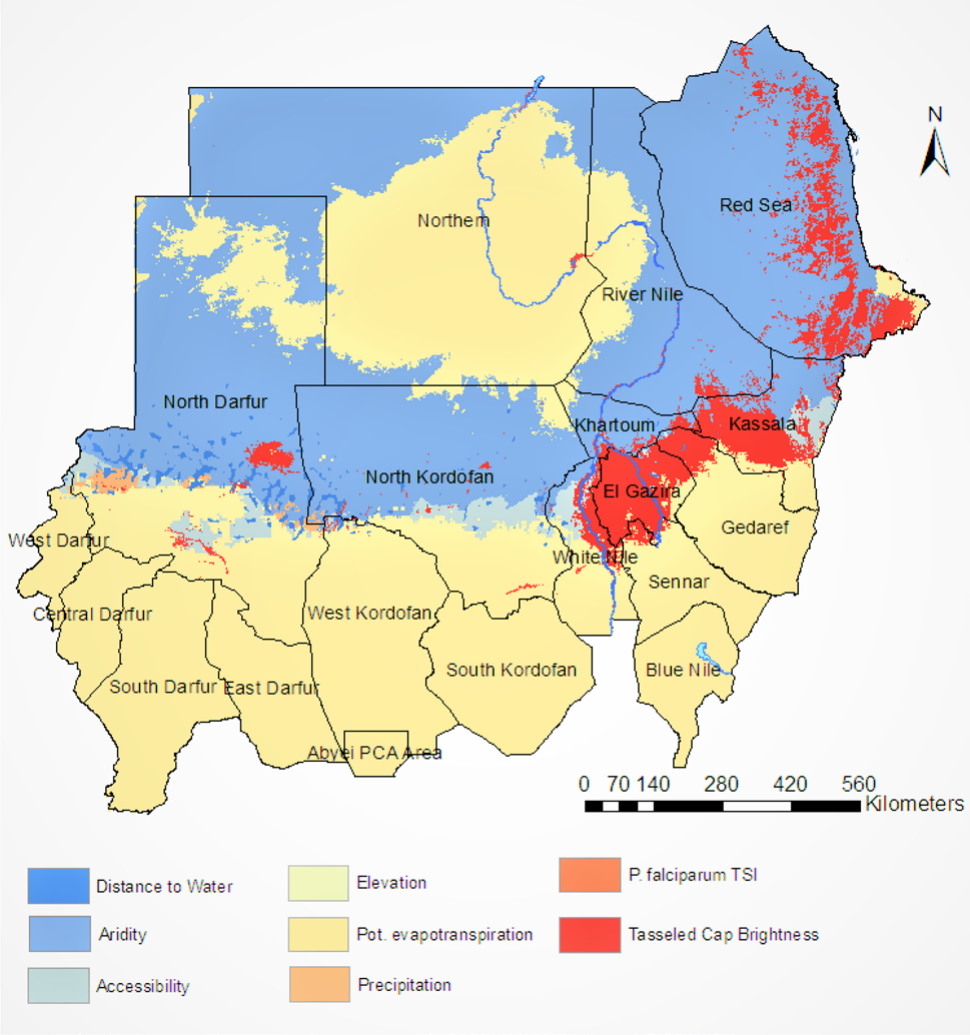
The covariates with the greatest positive influence in predicting the incidence rate, in each pixel, of *Plasmodium falciparum* malaria in Sudan. These covariates are associated with higher malaria incidence rate in the country.

The spatiotemporal Bayesian model used to produce the pixel-level endemicity maps includes a suite of fine-scale resolution environmental covariates as linear predictors that inform the estimation of the incidence rates. Each of these covariates has a slope that varies spatially to correspond to the best-fitting model. Figure 5presents the most impactful covariates for predicting pixel-level incidence rates. The impacts were estimated by multiplying the scaled pixel value of each covariate by its corresponding slope from the best fitting model. The largest leading positive covariates are illustrated in Fig. 5. In our model, the covariates most strongly correlated with *Plasmodium falciparum* incidence in Sudan were precipitation, aridity, tasselled cap brightness, and potential evapotranspiration.

#### *Plasmodium vivax* fine scale endemicity surface

**Figure 6:**
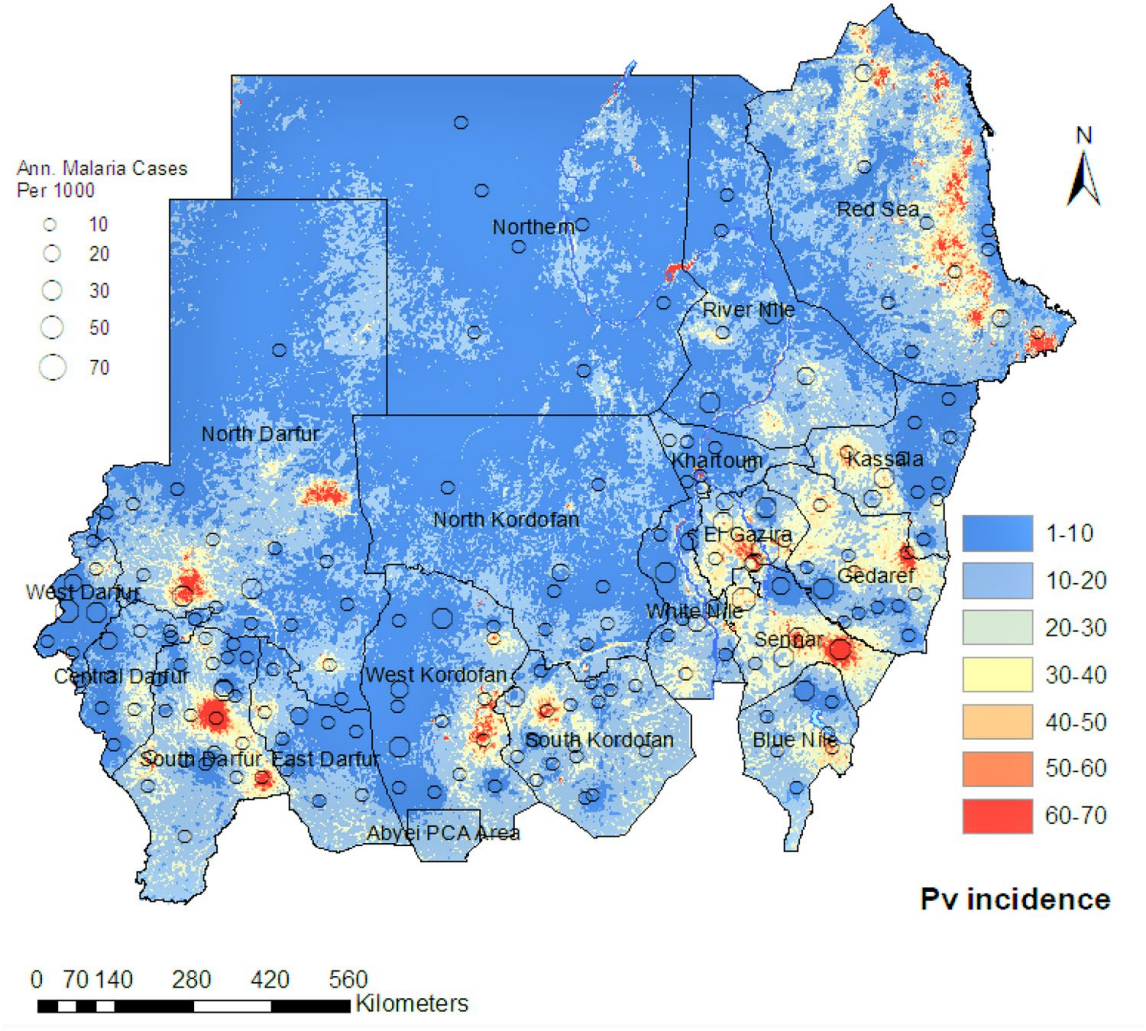
A fine-scale map ($1\times 1\,$km) of the incidence cases of the *P. vivax* malaria per 1000 in Sudan inferred based on our spatiotemporal Bayesian model fit to the routine surveillance data between 2017 and 2019. Hollow circles represent reported cases per year within each locality.

Figure 6 illustrates a fine-scale map ($1\times 1\,$km) of the *Plasmodium vivax* malaria infections in Sudan from our spatiotemporal Bayesian model, which we fit to the annual routine surveillance data from 2017 to 2019. Our model suggests that *Plasmodium vivax* infection is a newly unfolding public health issue in the country as it accounts for approximately $10\%$ of malaria cases in Sudan, This percentage is higher in the eastern and the central states relative to the rest of Sudan but, contrary to popular beliefs, this study demonstrates that *Plasmodium vivax* is present throughout Sudan^24,25,28–30^. It is also noteworthy that the *Plasmodium vivax* incidence rate in the Darfour region is comparable to the eastern and southeastern states bordering Ethiopia and Eritrea.

**Figure 7:**
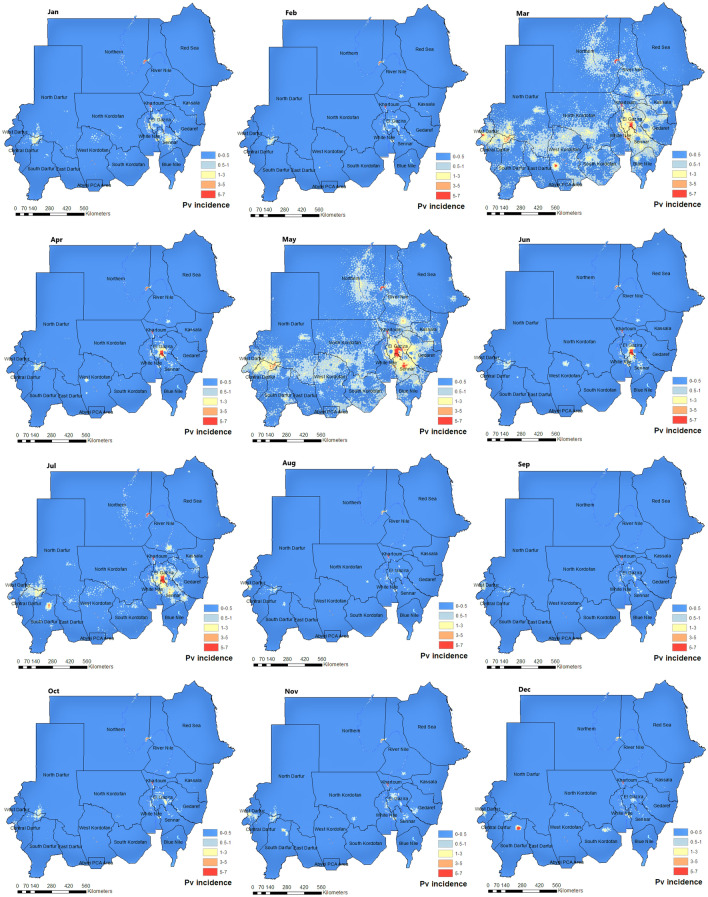
A fine-scale map ($1\times 1\,$km) of the incidence cases of the *Plasmodium vivax* malaria per 1000 in each calendar month in Sudan based on our spatiotemporal Bayesian model fit to the monthly routine surveillance data between 2017 and 2019. These maps were created in ArcGIS (https://www.arcgis.com) using the ArcMap package.

Figure 7 presents a fine-scale map ($1\times 1\,$km) of *Plasmodium vivax* incidence per calendar month in Sudan inferred based on our spatiotemporal Bayesian model fit to the monthly routine surveillance data between 2017 and 2019. The peak season of *Plasmodium vivax* malaria transmission occurs between March-and-May (see also Fig. 9) after the end of the *Plasmodium falciparum* peak season (July–November). Noteably, the transmission of *Plasmodium vivax* and *Plasmodium falciparum* in Sudan occur even during the dry seasons (March–June). These infections could be linked to the spread of a non-native malaria vector in Sudan, *Anopheles stephensi*^22^, which is known to thrive in urban and peri-urban settings and often breeds in man-made water containers^31^.

**Figure 8:**
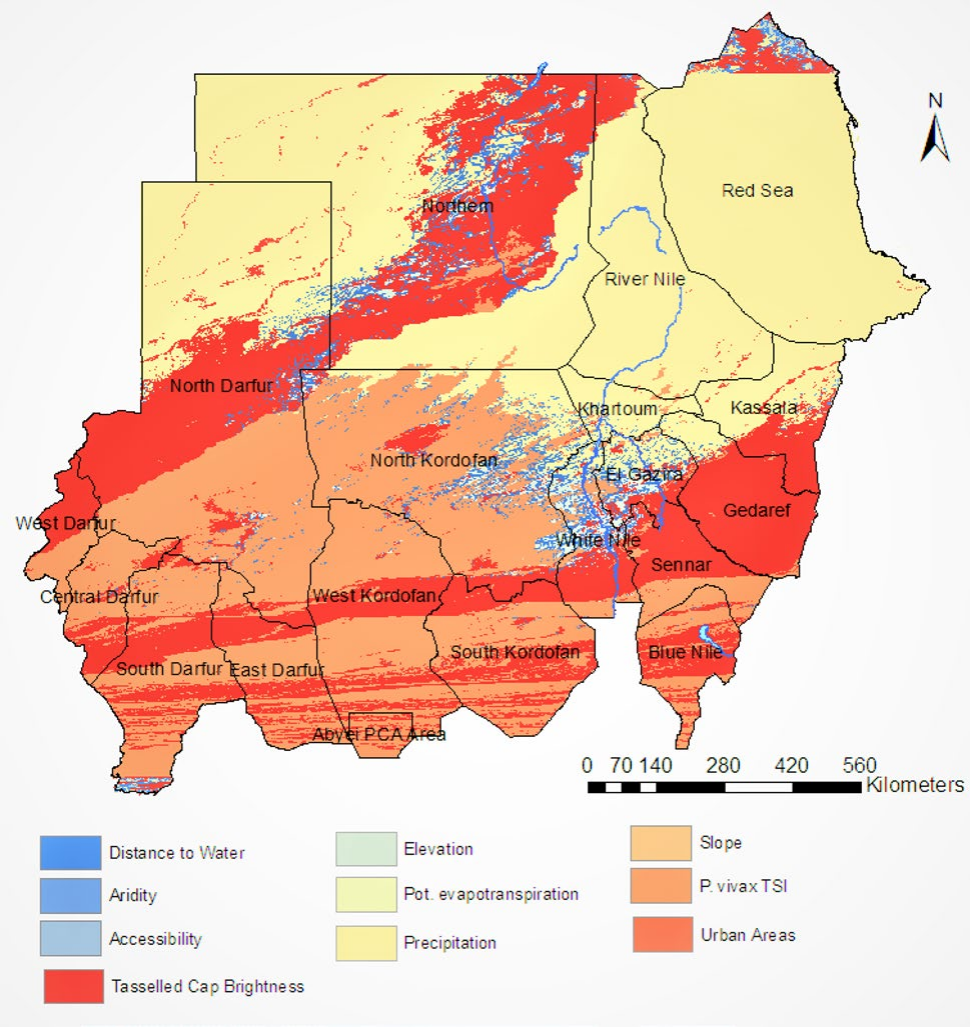
The most influential covariates for predicting the incidence rate of*Plasmodium vivax* in Sudan. These covariates are associated with leading the increase in the malaria incidence rate.

Similar to the *Plasmodium falciparum* endemicity analysis, the spatiotemporal Bayesian model used to produce the pixel-level *Plasmodium vivax* endemicity map included a suite of fine-scale resolution environmental covariates as linear predictors of the incidence rates. Fig. 8 presents the most influential covariates in the *Plasmodium vivax* incidence model, covariates that have positive coefficients in the fitted model are illustrated in this figure. The most important covariates within the model were precipitation, tasselled cap brightness, and *Plasmodium vivax* temperature suitability index.

## Discussion

**Figure 9:**
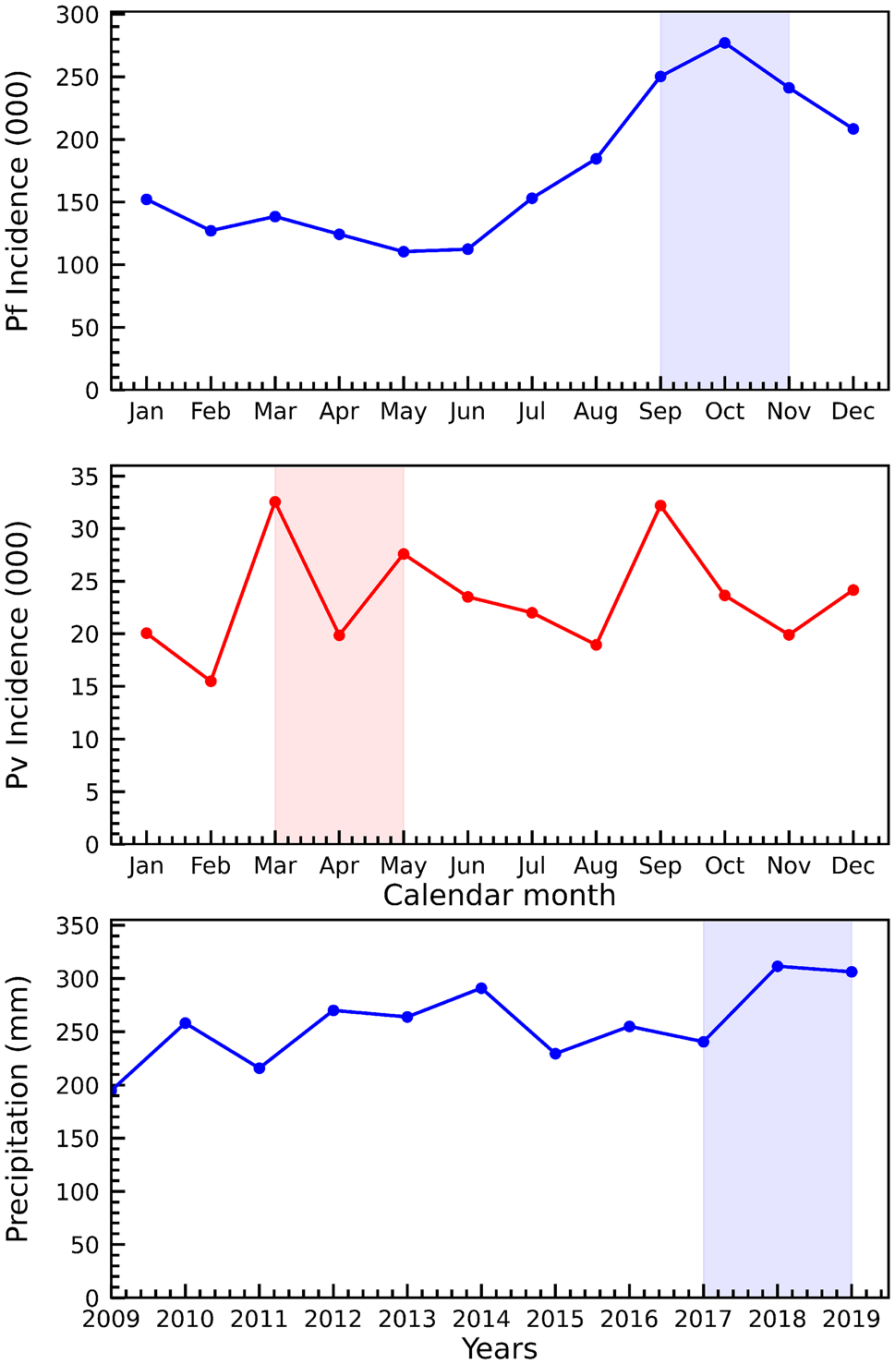
The seasonal variation of clinical species-specific malaria incidence in Sudan, *Plasmodium falciparum* (upper panel) and *Plasmodium vivax* (middle panel). The shaded regions represent the main peak of transmission of each species. The lower panel shows annual mean rainfall in Sudan between the year 2009 and 2019 and the shaded area highlights the modelled years. This figure was created in R (https://www.r-project.org/) using the ggplot package.

Malaria endemicity in Sudan exhibits a seasonal, inter-annual, and spatial heterogeneity, resulting from the interplay between climate variability (rainfall, temperature, and humidity) and anthropogenic context (population density, movement patterns, and human settlements). The main peak of malaria transmission for *Plasmodium falciparum* occurs shortly after the rainy season (July and early November) and a second, less intense peak of transmission occur at the beginning of winter (December to early February). In contrast, the main *Plasmodium vivax* transmission peak takes place between March and May, after the winter peak of *Plasmodium falciparum*. A second intense peak of *Plasmodium vivax* transmission occurs during the rainy season but lasts shorter than corresponding peak in *Plasmodium falciparum* (see Fig. 9).

The Ministry of Health in Sudan has identified six malaria epidemiological strata in the country. Those are seasonal, riverine, irrigated schemes, desert-fringe, urban malaria, and emergency and conflict zones malaria^32^. Our seasonality maps augment this classification of strata by adding a temporal dimension to this stratification. In Fig. 4, the transmission of *Plasmodium falciparum* remains high in Al-Gezira and Sennar states throughout the year. These states are both agricultural regions in Sudan with widespread irrigation infrastructure that provides suitable *Anopheles* breeding sites . Currently, only single rounds of IRS are implemented in Al-Gezira and Sennar annually for malaria control. Our analysis suggest that an alternate malaria control strategy may be beneficial in these states due malaria transmission throughout the year. Possible strategies for these areas include increasing coverage of ITNs and Larval Source Management (LSM). The seasonality maps of *Plasmodium vivax* (Fig. 7) show that Khartoum state (i.e., urban area and the capital of Sudan) has the most stable and highest *Plasmodium vivax* malaria transmission in Sudan regardless of month. Other states with continuous transmission of *Plasmodium vivax* malaria are central Darfour and Al-Gezira states. Our *Plasmodium vivax* seasonality maps reveal new malaria epidemiological strata, which differ from to the Ministry of Health classifications. Our analysis shows that states like the Northern state (desert ecology) and the Red Sea had low transmission of *Plasmodium falciparum* and *vivax*. These findings support the most recent National Malaria Strategic Plan (NSP) for 2021–2025, which identifies these states as potential targets for elimination.

Sudan has unreliable and erratic rainfall, with great differences between the northern and southern parts for the country. In the northern regions, little to no precipitation falls annually ($< 50$ mm per year), in the central regions annual precipitation ranges from 200 to 700 mm per year, and in some southern regions more than 1500 mm of precipitation fall each year. Sudan’s precipitation is concentrated in the rainy season that occurs between July and September. Unusually intense rainfall in 2018 and 2019 led to widespread flooding, destruction of housing, and an increase in malaria infections^9^. Such events are likely to increase in frequency as a consequence of climate change (see Fig. 9), which will have important implications for malaria in Sudan. The floods in 2018 and 2019 caused substantial increased the population’s vulnerability to infectious diseases while also trapping water destroyed building thus providing additional breeding sites for malaria vectors. The destruction associated with the flooding also impacted health facilities and healthcare providers themselves, thereby reducing access to healthcare services among the severely damaged communities.

The recent emergence and the yet-unknown distribution of *Anopheles stephensi* in Sudan^18^, may also have considerably contributed in the recent epidemics of malaria that have occurred in different regions of Sudan^15–17^. Our results show an increase of *Plasmoidum vivax* malaria cases in Urban areas between 2017 and 2019, particularly in the Red Sea, Kassala, Gedarif and Khartoum states. *Anopheles stephensi* mosquitoes have been detected in each of these states^17^, suggesting a possible link with *Plasmodium vivax* malaria cases. A similar scenario occurred in Djibouti in 2012 when serious epidemics of malaria took place in urban settings throughout the country and subsequent entomological investigations confirmed the first appearance *Anopheles stephensi* in Africa^33,34^. Other studies have warned about the serious threat of malaria epidemics brought by the *Anopheles stephensi* in the urban settings of Africa^33,35^, as this invasive vector was experimentally proven to be competent in transmitting both *Plasmodium falciparum* and *Plasmodium vivax*^20^. Recent studies have indicated that this vector’s distribution in Africa is expanding rapidly, but remains undetected in some areas due to the limited surveillance capacity, inability to apply molecular techniques for species identification, and the poor implementation of International Health Regulations (IHRs 2005)^19,21,36^. Lastly, evidence-based habitat suitability models suggest that Sudan is entirely within the zone that is more likely to be invaded by *An. stephensi*^37^.

The socioeconomic and environmental changes driven by armed conflicts increase the vulnerability of the displaced communities including migrants, refugees, and internally displaced persons^38^. For instance, dengue fever was found only in Eastern Sudan since the 1900s, but this disease has emerged in the Western region of the country, namely Darfour in 2014, when this region experienced repeated conflicts^39^. Similarly, other vector-borne viral diseases including the West Nile virus and Crimean-Congo hemorrhagic fever (CCHF) emerged in Darfour in 2015–2016^40^. Extreme weather events such as the flooding in Sudan in 2018 and 2019 also have disproportionate epidemiological impacts in areas of humanitarian crisis due to very limited services for healthcare and environmental vector control.

In Sudan, the control of malaria transmission is multifaceted problem and is not only limited to the socioeconomic and environmental factors. Recent studies have found evidence suggesting the inaccuracy of rapid diagnostic tests (RDTs) results due to histidine-rich protein 2/3 (pfhrp2/3) gene deletion in *Plasmodium falciparum* isolates in Sudan^41–43^. The deletions of the pfhrp2/3 genes in *Plasmodium falciparum*-infected populations leads to false-negative diagnoses of malaria based on RDT^44^, delaying the immediate administration of antimalarial drugs, and consequently the spreading of such genotypes from untreated patients to the entire population. As such, populations of *Plasmodium falciparum* containing these variants of the pfhrp2/3 pose a significant threat to ongoing efforts to control and eliminate malaria in Sudan^45^. There has been no country-wide survey with which the extent of this gene deletion in the Sudan, which poses a further challenge to the Malaria Control Program in the country. Another challenge in characterising malaria in Sudan is the lack of a WHO non-hrp2 test that distinguishes between *Plasmodium falciparum* and *Plasmodium vivax*^6,8^.

There are reasons this research study should be viewed in the context of some limitations. First, the health facility dataset is not designed for the specific purpose of high-resolution spatial predictive analysis, and a high degree of prediction uncertainty exists for areas in the country where data is sparse. The inequality in the distribution of health facilities across the country also leads to the under-reporting of malaria, especially in remote areas which in terms affects the model results. Another source of uncertainty in this research arises from our spatiotemporal model approach, detailed below. This model links ecological-level effects (i.e., measured at the level of the aggregation unit, the health facility in this case, where incidence is estimated) to individual-pixel level effects. The difference in spatial resolution between individual-pixel level effects and ecological-level effects at the health facility introduces some uncertainty in the predicted incidence at pixel levels that increases in locations with fewer health facilities. Even though this research has limitations, the endemicity maps presented in this manuscript will be of great use to the Ministry of Health in strategic planning.

In summary, our results show a high incidence of malaria in the Republic of Sudan in the years between 2017 and 2019, with recent increases coincident with flooding events in 2018 and 2019. Spatial and species-specific heterogeneity at sub-national and local levels largely reflects underlying topological, climatic, and socioeconomic factors, including population migration and displacement. We recommend strategic and targeted interventions in states with low endemicity like the Northern and the Red Sea states where the elimination of malaria is plausible in the short term. Elsewhere, our endemicity maps provide a potential resource for guiding intervention strategies and identifying deficiencies in malaria surveillance. In regions with hyperendemicity, such as the central region (Khartoum area), the western and south-western regions (Darfour and south Kordofan), and the eastern regions (Sennar, White Nile and Al-Gezira), we strongly recommend the scale-up of all the available interventions,particularly during and after the raining season.

## Methods

The main dataset used in this work was provided by the National Malaria Control Program (NMCP) of the Republic of Sudan. The data consist of monthly malaria confirmed cases in each health facility (n=461) in the country, as confirmed by either RDT or microscopy, and containing information on the species type, *Plasmodium falciparum*, *Plasmodium vivax*, or others including mixed infection of both *Plasmodium falciparum* & *Plasmodium vivax* or *Plasmodium ovale*). The reporting period spans 3 years between January 2017 and December 2019, with a $70\%\,$ overall completeness. In addition to the health facility dataset, we use a suite of ($1\times 1\,$km resolution) environmental covariates as independent variables in our statistical model. These covariates include the accessibility to cities^46^, precipitation^47^, Potential evapotranspiration^48^, aridity index^48^, the shuttle radar topography mission elevation measurement^49^, distance to permanent and semi-permanent sources of water^50^, tasselled-cap brightness^51^, land surface temperature (night-time)^52^, slope^49^, and classified land cover (urban/rural areas)^53^. Each of these covariates has been used extensively in previous spatial malaria models, and has demonstrated statistical association with varying malaria risk^54–56^.

The main goal of this paper is to generate high-resolution, monthly estimates of the malaria incidence rate for *Plasmodium falciparum* and *Plasmodium vivax* malaria in Sudan between 2017 to 2019. To calculate the malaria incidence rates at each health facility, we first estimate the population of patients likely to seek treatment in each facility (i.e., the catchment population). This population is derived based on the time it takes to travel to each health facility, using the friction surfaces^46^. In this process, we first estimate the travel time to each of the health facilities for each pixel. We then model the probability of patients in each pixel seeking treatment in each health facility as proportional to the inverse square of the time it takes to reach that health facility^55,56^. Our approach to the estimation of the catchment populations is similar to the gravity model in which both the availability and accessibility to health facilities across defined spatial units are considered^57,58^. The resulting catchment population for each health facility ($H_{k}$) is the summation the treatment-seeking probability from each pixel (*l*) in our grid to that health facility $H_{k}$ multiplied by the treatment seeking-adjusted population of that pixel ($P_{l}$).

1 $$\begin{aligned} \Sigma _{l=1}^{N}\,P_{l} \times Prob\,(Pixel_{l} \longrightarrow H_{k}) \end{aligned}$$

The fraction of the population that will seek treatment in any health facility ($P_{l}$) is estimated using a logistic function of travel time to any health facility following established approaches in^55,59^. We use the WorldPop population surfaces to estimate the catchment population for each year between 2017 and 2019^60^.

After calculating the catchment population, we estimate the incidence rate at each health facility in the country to use as our response variable. We associate the facility-level incidence rate with the environmental covariates described above within a Bayesian spatiotemporal model to produce both annual and monthly incidence rate surfaces at a $1\,$km$\,\times \,1\,$km spatial resolution. We model malaria incidence cases as a Poisson process, i.e., malaria incidence cases at a location *i* and time *t* is given by:

2 $$\begin{aligned} \eta _{i\,t} \sim poisson\,(n_{i} \times \tau _{i\,t}) \end{aligned}$$

where $\eta _{i\,t}$ is the observed malaria incidence, $n_{i}$ is the catchment population and $\tau _{i\,t}$ is the malaria incidence rate at location *i* and time *t*. The logarithmic malaria incidence rate is modeled with the following linear regression predictors:

3 $$\begin{aligned} log(\tau _{i,t})= \alpha + \Sigma _{z=1}^z \beta _z X_{z,i,t} + f(s_{i},t) \end{aligned}$$

where $\beta$ is a matrix of covariate coefficients, $\alpha$ is the intercept, *X* is a matrix of *z* covariates, and *f*(., *t*) is a realisation (for each year, or each month in case of the seasonal incidence rate) of a Gaussian process over space with a zero-mean Gaussian Markov random field (GMRF) and a Matérn covariance function. The covariance function is parameterised by, $\sigma$ the marginal standard deviation as well as $\rho$ (the distance beyond which correlation becomes negligible). We exploit the Gaussian Markov Random Field approximation^61^ to fit our incidence model using a combination of the Template Model Builder package^62^ as well as INLA package^63^ in R language^64^. We produce associated uncertainty estimates from 2000 samples drawn from a Laplace approximation of the posterior and quantify those using the standard deviation and the interquartile range of the posterior distribution, as well as the exceeding and non-exceeding probabilities. We derive the prediction of the incidence surfaces for *Plasmodium falciparum* and *Plasmodium vivax* in Sudan separately, a model that jointly considers the malaria endemicity of both species in Sudan is under preparation and will appear in a separate report. We have compiled all the model validation results and goodness of fits in tables as well as diagnostic figures that will help the reader assess the quality of this converged model in the Supplementary material.

## Supplementary Information

Supplementary Information.

## Data Availability

The routine surveillance data are the property of the Sudanese National Malaria Control Program and the Sudanese Ministry of Health. All covariates used in this paper are available on the Malaria Atlas Project website https://malariaatlas.org/^54^.
