# Supplementary material for: Spatiotemporal mapping of malaria incidence in Sudan using routine surveillance data
Source: Sci Rep. 2022 Aug 18;12:14114. doi: 10.1038/s41598-022-16706-1 (PMC9387890; doi:10.1038/s41598-022-16706-1)
Supplement: Supplementary file 1 — Supplementary Information. [file 41598_2022_16706_MOESM1_ESM.pdf]

Spatiotemporal mapping of malaria incidence in Sudan using routine surveillance data

Ahmed Elagali[1,2*], Ayman Ahmed[3,4,5], Nada Makki[6], Hassan Ismail[7], Mark Ajak[8], Kefyalew Addis Alene[1,9], Daniel J. Weiss[1,9], Abdalla Ahmed Mohammed[10], Mustafa Abubakr[11], Ewan Cameron[1,9], Peter Gething[1,9], and Asmaa Elagali[2]

[1]Telethon Kids Institute, Perth, Western Australia, Australia
[2]Department of Zoology, Omdurman Islamic University, Khartoum, Sudan
[3]Institute of Endemic Diseases, University of Khartoum, Khartoum 11111, Sudan
[4]Swiss Tropical and Public Health Institute (Swiss TPH), 4123 Allschwil, Switzerland
[5]University of Basel, Petersplatz 1, CH 4001, Basel, Switzerland
[6]Health Information Management and Statistics, Federal Ministry of Health, Khartoum, Sudan
[7]Neglected Tropical Diseases Control Division, Federal Ministry of Health, Khartoum, Sudan
[8]Preventive Health Services, National Ministry of Health, Juba, South Sudan
[9]Faculty of Health Sciences, Curtin University, Perth, Western Australia, Australia
[10]Diseases Control Directorate, Federal Ministry of Health , Khartoum, Sudan
[11]Department of the Integrated Vector Management (IVM), Federal Ministry of Health, Khartoum, Sudan
[*]ahmed.elagali@telethonkids.org.au

Supplementary material

Both the *Plasmodium falciparum* and *Plasmodium vivax* models have converged and produced reasonable estimates for the pixel level malaria endemicity in Sudan. In this supplementary material, we provide the reader with all the model validation results and goodness of fits in tables as well as diagnostic figures that will help assess the quality of these models.

Covariates	Short Definition
AI	Aridity index; an indicator for the dryness of land.
Urban Areas	A classification of land cover to urban or rural area.
Precipitation	An indicator for rainfall and drought of land.
TCB	Tasselled cap brightness;a measure of land reflectance.
TSI Pf	An index that measures the temperature suitability for sporogony & vector survivorship for P. falciparum.
TSF Pv	An index that measures the temperature suitability for sporogony & vector survivorship for P. vivax.
Accessibility	Distance to cities with population $> 50,000$.
PET	Potential evapotranspiration; a measure of evaporation occurring in the air.
Elevation	A measurement for land elevation obtained by the shuttle radar topography mission (SRTM).
Slope	GIS-derived surface calculated from SRTM elevation surface.
Night lights	Index that measures the presence of lights from towns, cities and other sites with persistent lighting.
Distance to water	GIS-derived surface that measures distance to permanent and semi-permanent water sources.

Table 1. Short definitions for the covariates used in the *Plasmodium falciparum* and *Plasmodium vivax* incidence models. References for these environmental covariates are available in the main manuscript.

Parameter	mean	Standard Deviation	CI
Intercept	-4.696	0.194	-5.078,-4.315
Distance to water	0.031	0.137	-0.238,0.301
AI	-0.755	0.409	-1.557,0.047
Access to HF	-0.456	0.312	-1.069,0.157
Elevation	-0.274	0.189	-0.645,0.098
PET	1.492	0.327	0.850,2.134
Precipitation	1.670	0.501	0.687,2.653
Slope	-0.133	0.134	-0.397,0.131
TSI Pf	0.302	0.216	-0.121,0.725
Urban Areas	-1.458	0.679	-2.791,-0.127
TCB	-0.863	0.211	-1.278,-0.448
Night time lights	0.044	0.127	-0.206,0.294
θ_1 spatial field	-4.886	0.215	-5.334,-4.491
θ_2 spatial field	3.127	0.156	2.832,3.445
ρ spatial field	0.605	0.037	0.535,0.679

Table 2. Mean, standard deviation and 95% credible intervals for the *Plasmodium falciparum* incidence model parameters.

Model parameters Tables & Goodness of Fit

The posterior mean, standard deviation and 95% credible interval for all the *Plasmodium falciparum* model parameters are shown in Table 2 and for the *Plasmodium vivax* are shown in Table 3. Figures 2-4 illustrate the posterior distribution of both the fixed parameters and hyperparameters for both the *Plasmodium falciparum* and *Plasmodium vivax* models. Figure 1 illustrates a comparison between the observed versus predicted incidence rates values for the *Plasmodium falciparum* and *Plasmodium vivax* models. The data points used in these two plots constitute 10% of the observed data-set, the other 90% were used to train the model.

Annual risk maps

The annual clinical incidence estimates for three different quartile ranges (25%,68% and 95%) for the *Plasmodium falciparum* model are shown in Figure 5 as well as the *Plasmodium vivax* model are shown in Figure 6.

Monthly risk maps

The monthly maps of clinical incidence estimates for three different quartile ranges (25%,68% and 95%) for the *Plasmodium falciparum* model are shown in Figures 7, 8 and 9 as well as the *Plasmodium vivax* model are shown in Figures 10, 11 and 12.

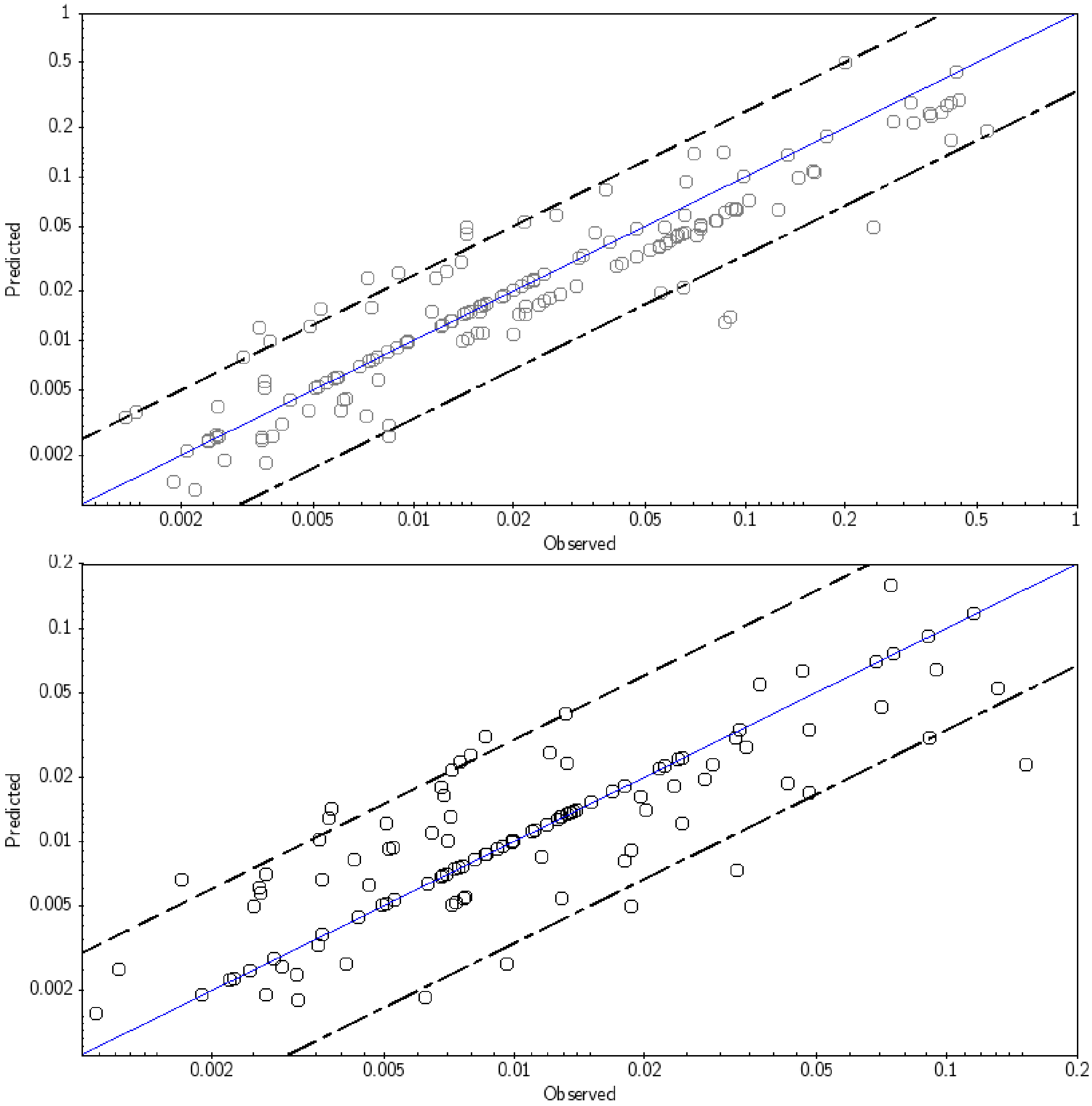

Figure 1. Observed versus predicted incidence rates values for the *Plasmodium falciparum* (upper panel) and *Plasmodium vivax* (lower panel) models. The blue line marks the equality $y = x$, whereas the dashed lines mark the 68 th percentile. These figures were created in R (https://www.r-project.org/) using the ggplot package

Parameter	mean	Standard Deviation	CI
Intercept	-6.580	0.195	-6.963,-6.198
Distance to water	0.003	0.136	-0.265,0.270
AI	-0.598	0.438	-1.455,0.268
Access to HF	-0.772	0.303	-1.367,-0.177
Elevation	0.011	0.200	-0.381,0.405
PET	0.835	0.341	0.165,1.504
Precipitation	0.499	0.463	-0.418,1.404
Slope	0.192	0.219	-0.239,0.622
TSI Pv	0.365	0.209	-0.045,0.775
Urban Areas	0.233	1.129	-1.991,2.447
TCB	-0.280	0.232	-0.738,0.174
Night time lights	-0.027	0.367	-0.748,0.693
θ_1 spatial field	-3.437	0.130	-3.696,-3.185
θ_2 spatial field	1.652	0.144	1.374,1.940
ρ spatial field	0.368	0.085	0.196,0.529

Table 3. Mean, standard deviation and 95% credible intervals for the *Plasmodium vivax* incidence model parameters.

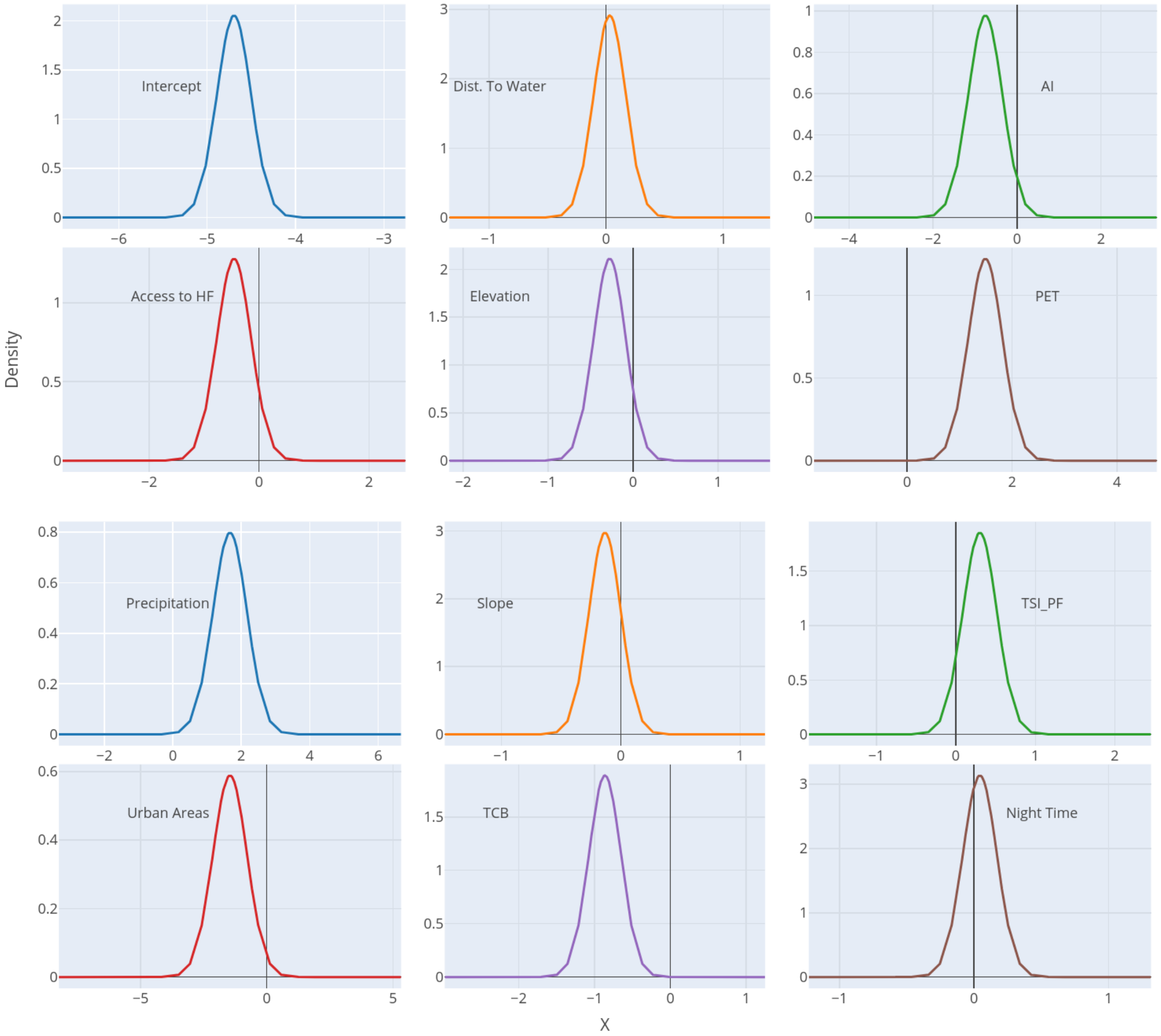

Figure 2. Posterior distributions of the *Plasmodium falciparum* incidence model fixed effects. These figures were created in R (https://www.r-project.org/) using the ggplot package

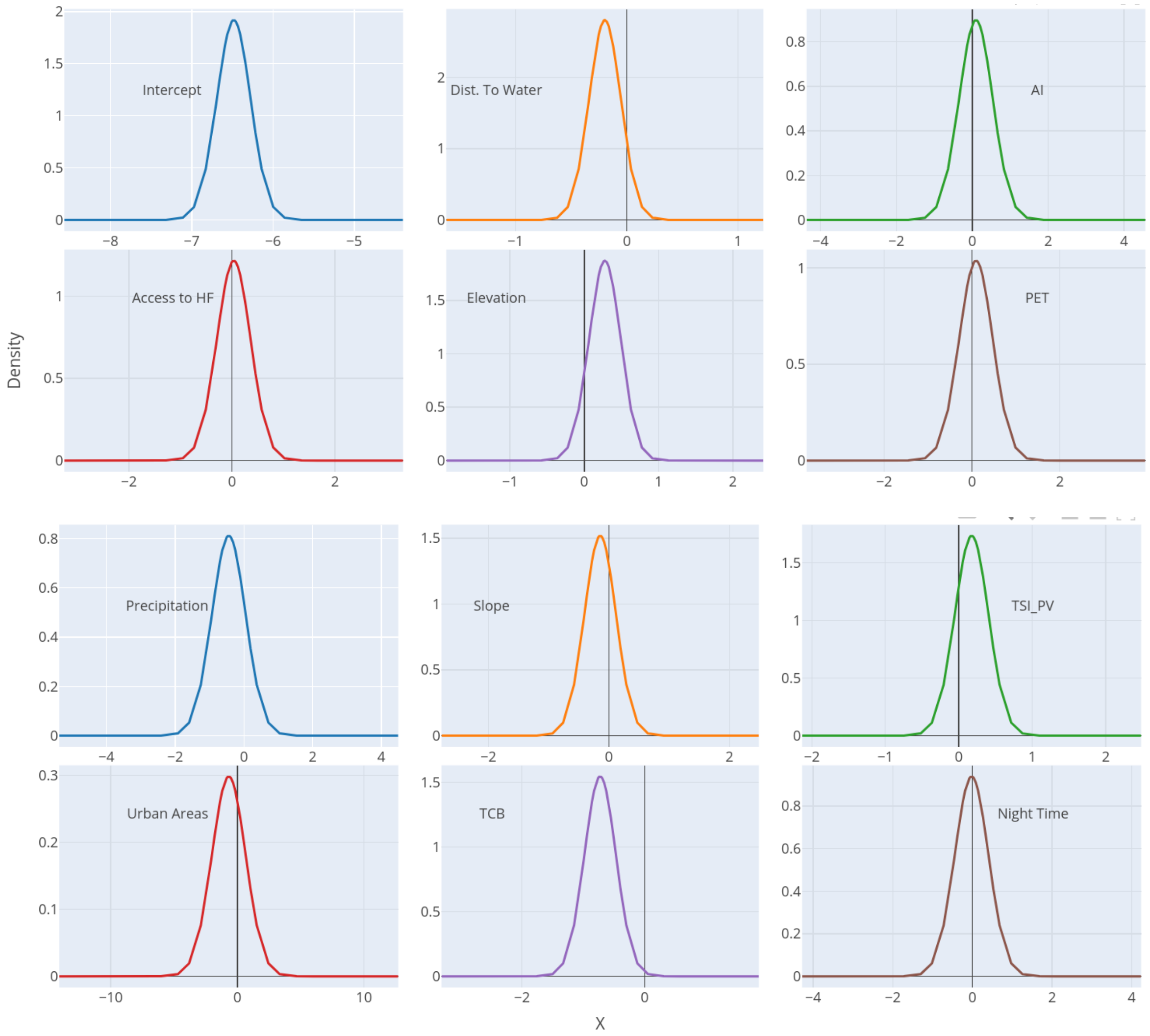

Figure 3. Posterior distributions of the *Plasmodium vivax* incidence model fixed effects. These figures were created in R (`https://www.r-project.org/`) using the ggplot package

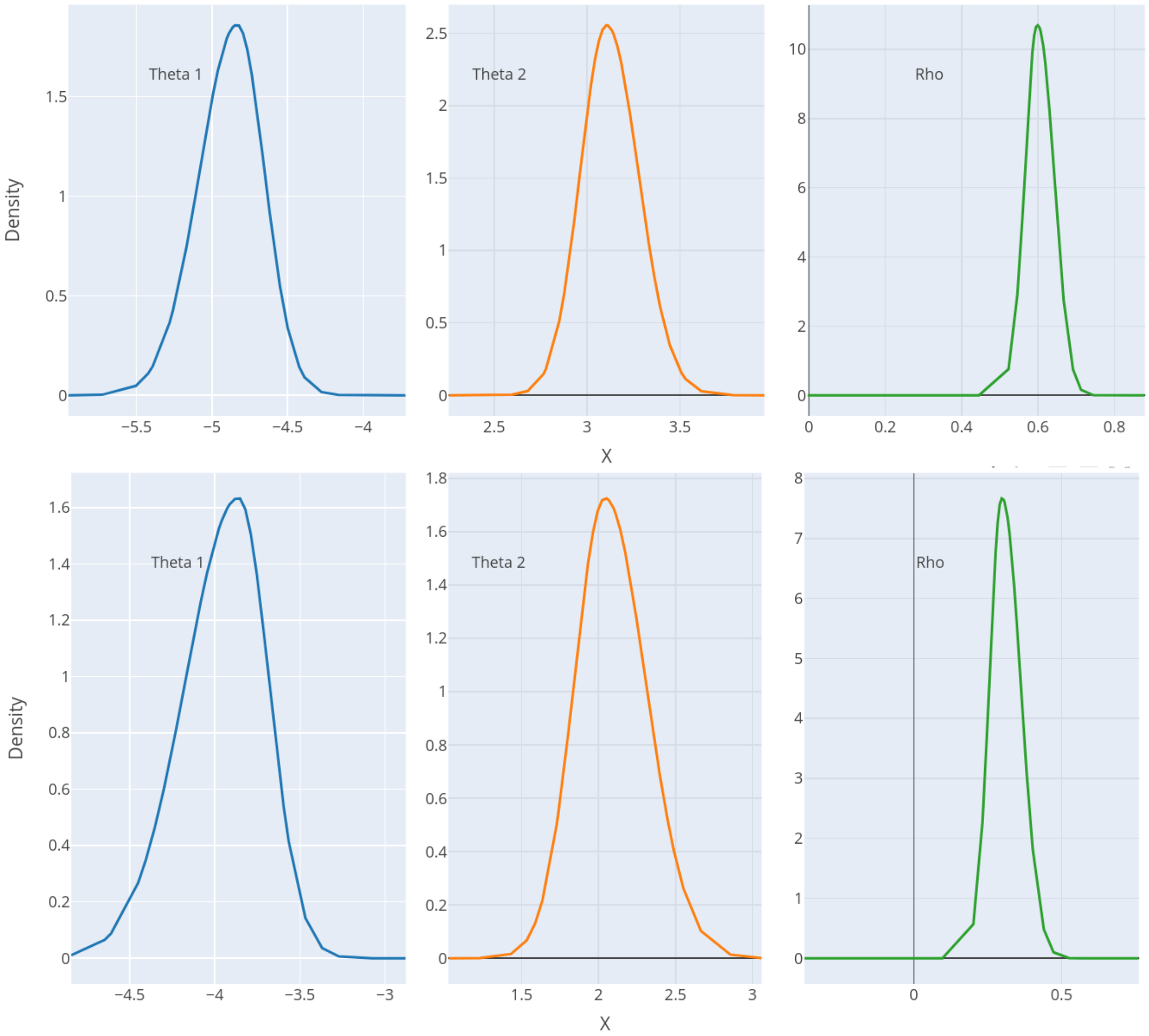

Figure 4. Posterior distributions of the *Plasmodium falciparum* (upper panel) and *Plasmodium vivax* (lower panel) incidence model hyperparameters. These figures were created in R (`https://www.r-project.org/`) using the ggplot package

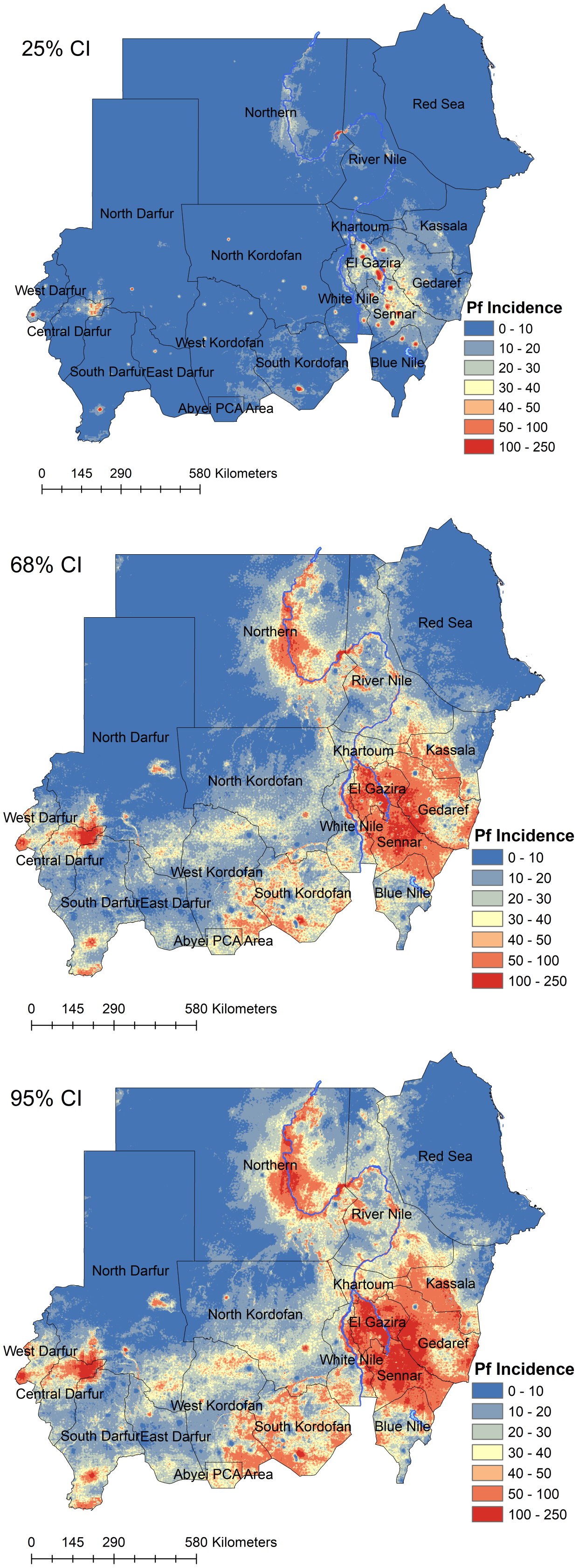

Figure 5. A fine-scale map (1×1 km) of the estimated 25%, 68% and 95% credible interval of the annual clinical incidence cases of *Plasmodium falciparum* malaria per 1000 in Sudan for years 2017-2019. These figure were created in ArcGIS (https://www.arcgis.com) using the ArcMap package.

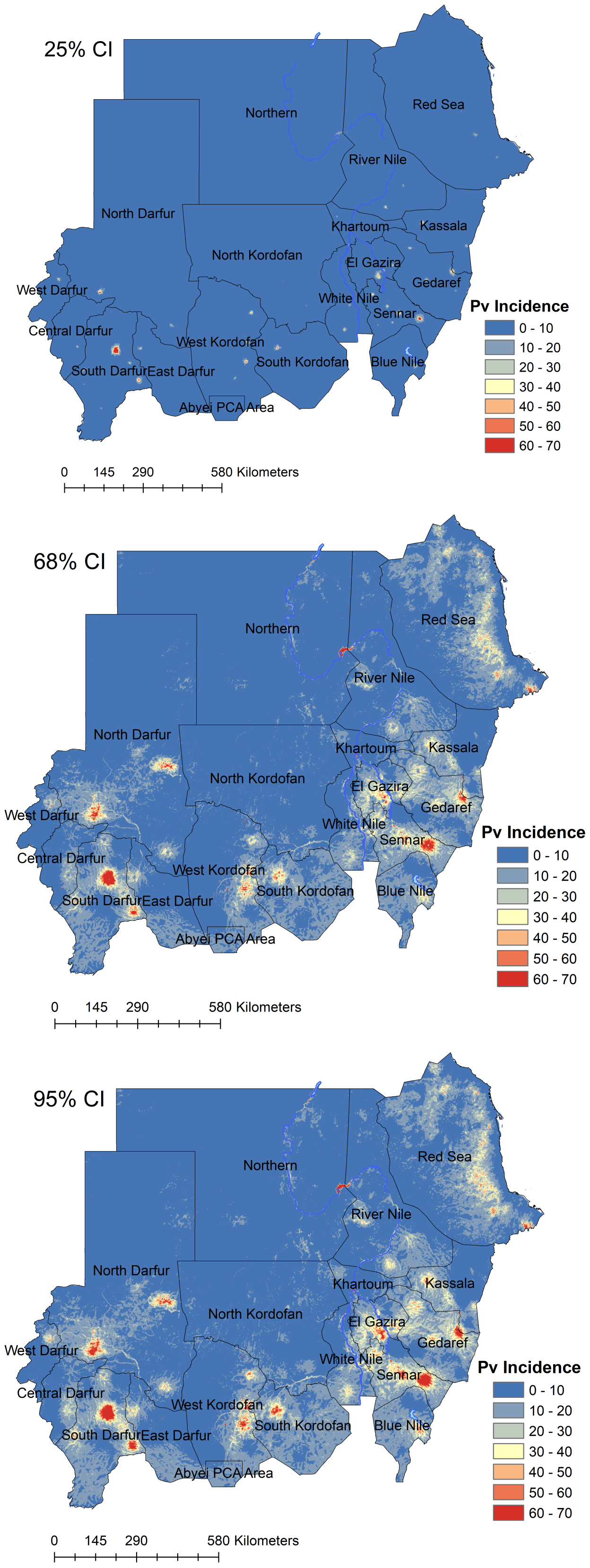

Figure 6. A fine-scale map (1×1 km) of the estimated 25%, 68% and 95% credible interval of the annual clinical incidence cases of *Plasmodium vivax* malaria per 1000 in Sudan for years 2017-2019. These figure were created in ArcGIS (`https://www.arcgis.com`) using the ArcMap package.

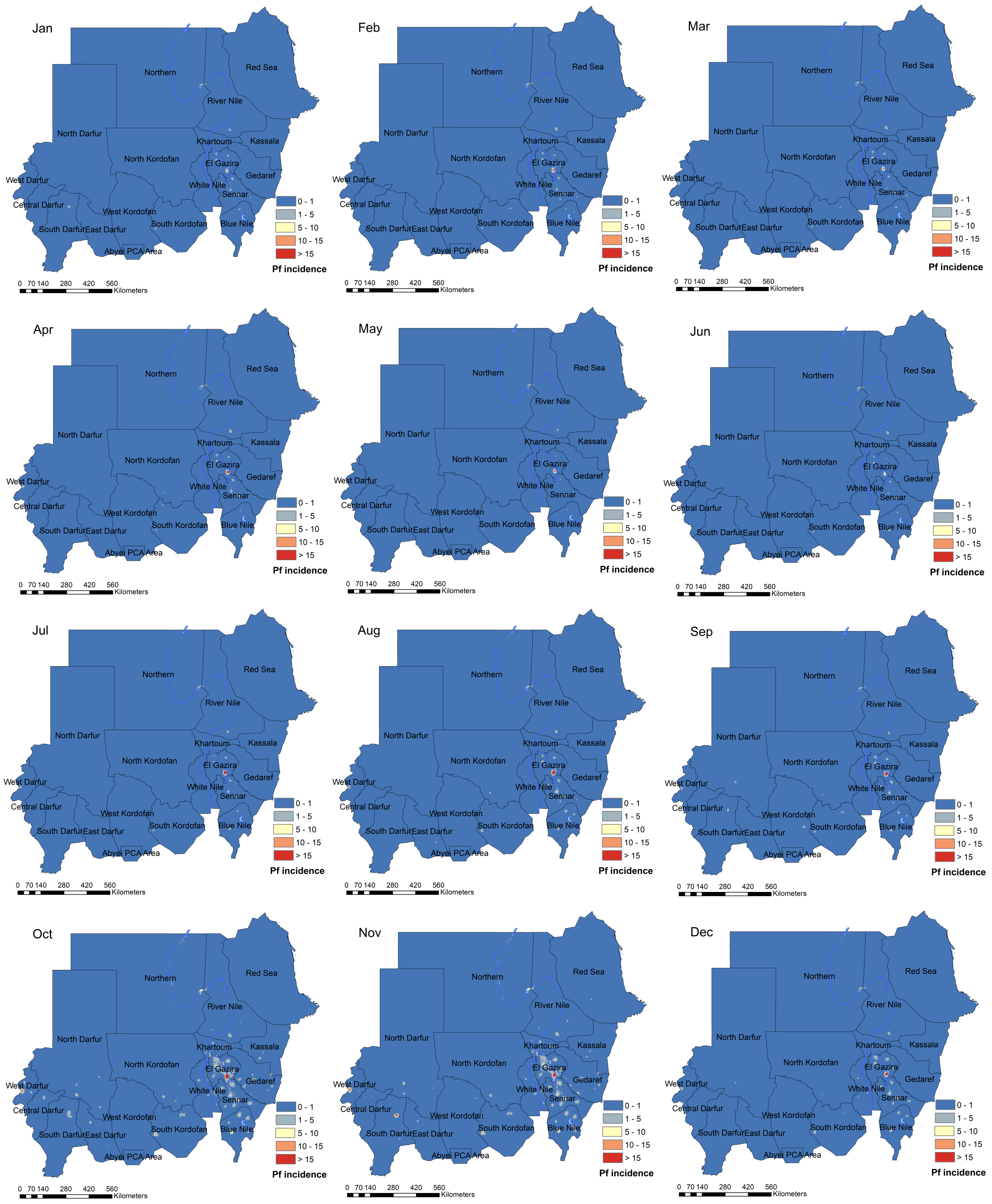

Figure 7. A fine-scale map (1 × 1 km) of the 25% credible interval of the incidence cases of *P. falciparum* malaria per 1000 in each calendar month in Sudan inferred based on our spatiotemporal Bayesian model fit to the monthly routine surveillance data between 2017-2019. These figure were created in ArcGIS (https://www.arcgis.com) using the ArcMap package.

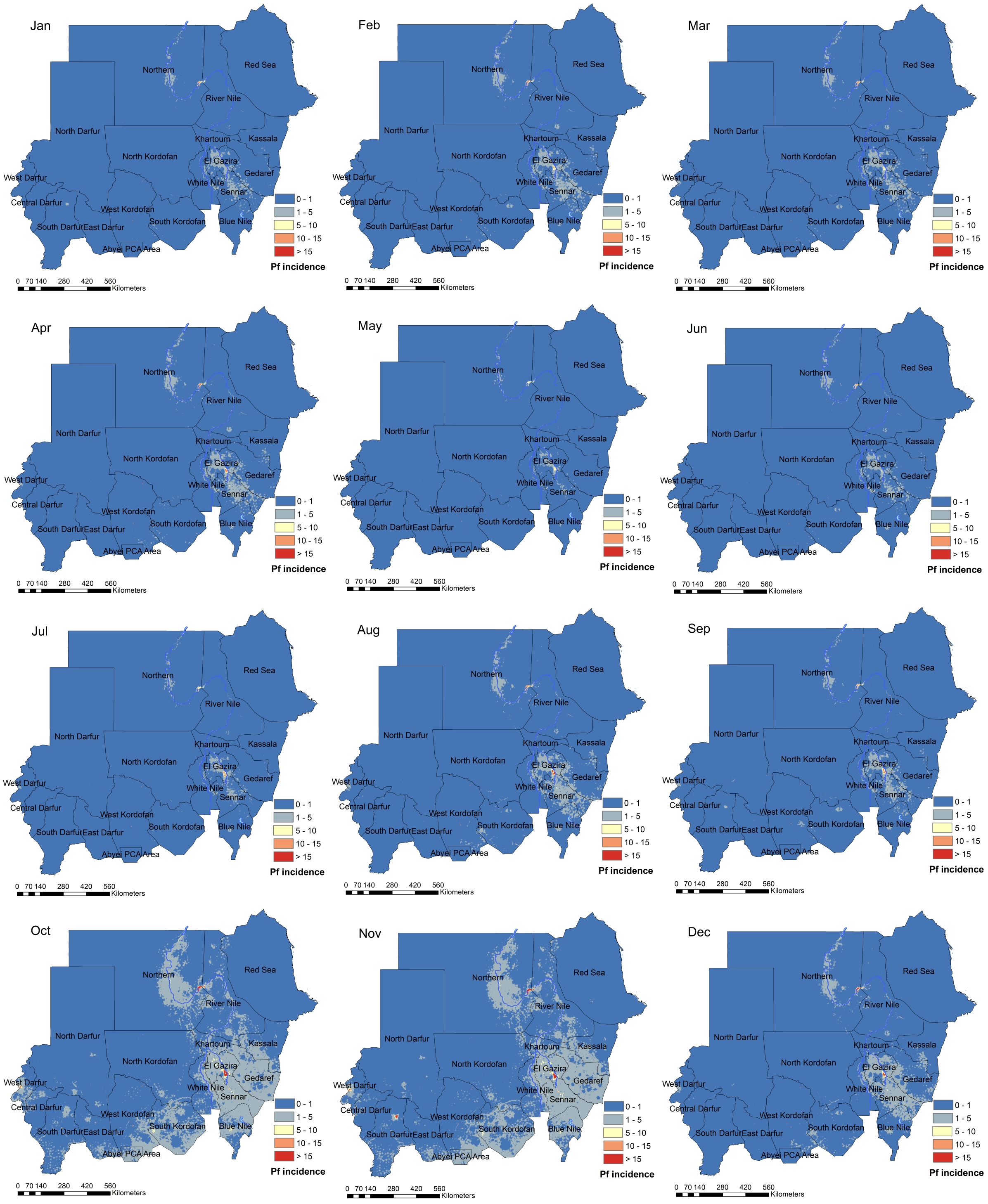

Figure 8. A fine-scale map (1×1 km) of the 68% credible interval of the incidence cases of *P. falciparum* malaria per 1000 in each calendar month in Sudan inferred based on our spatiotemporal Bayesian model fit to the monthly routine surveillance data between 2017-2019. These figure were created in ArcGIS (`https://www.arcgis.com`) using the ArcMap package.

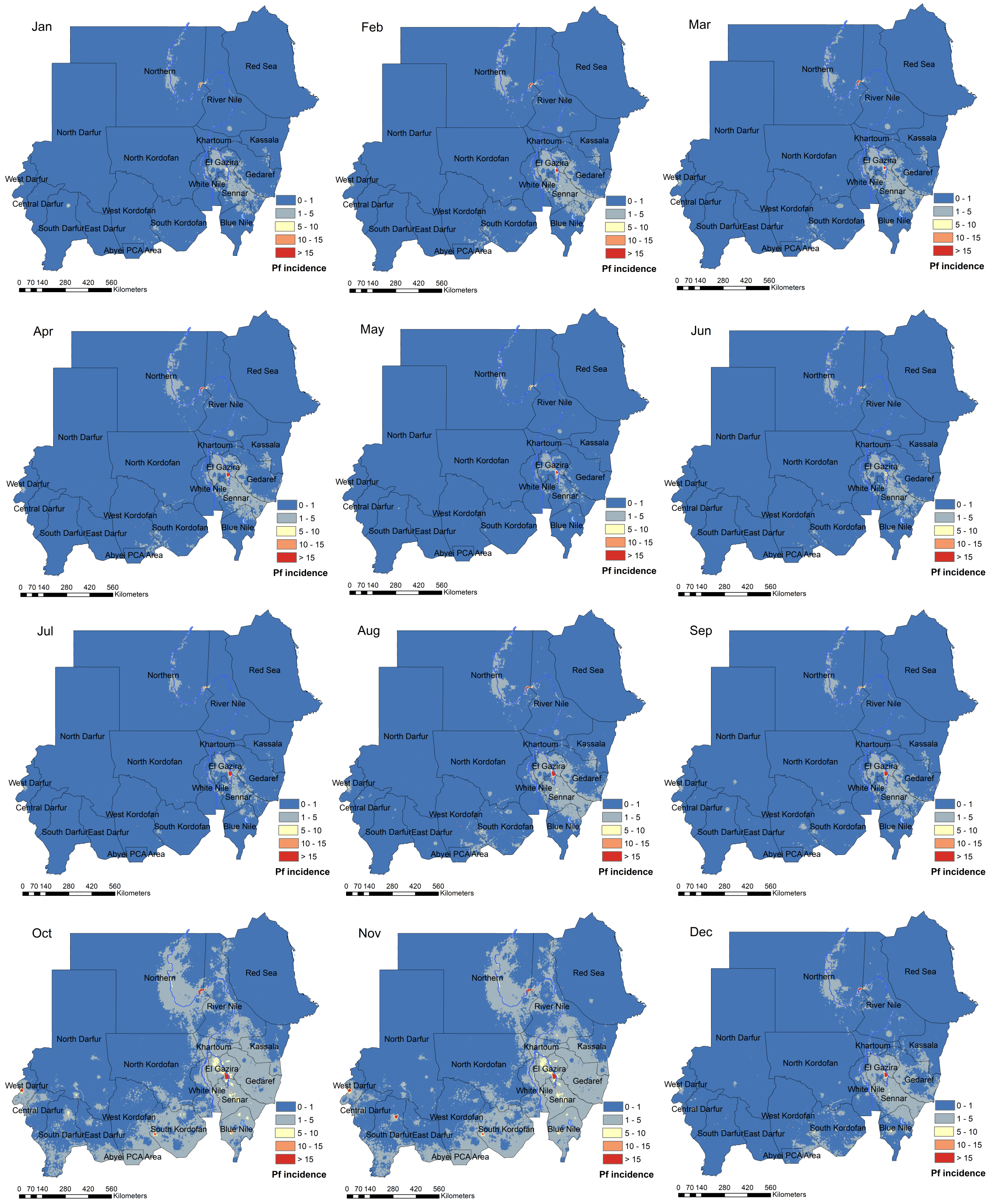

Figure 9. A fine-scale map (1 × 1 km) of the 95% credible interval of the incidence cases of *P. falciparum* malaria per 1000 in each calendar month in Sudan inferred based on our spatiotemporal Bayesian model fit to the monthly routine surveillance data between 2017-2019. These figure were created in ArcGIS (https://www.arcgis.com) using the ArcMap package.

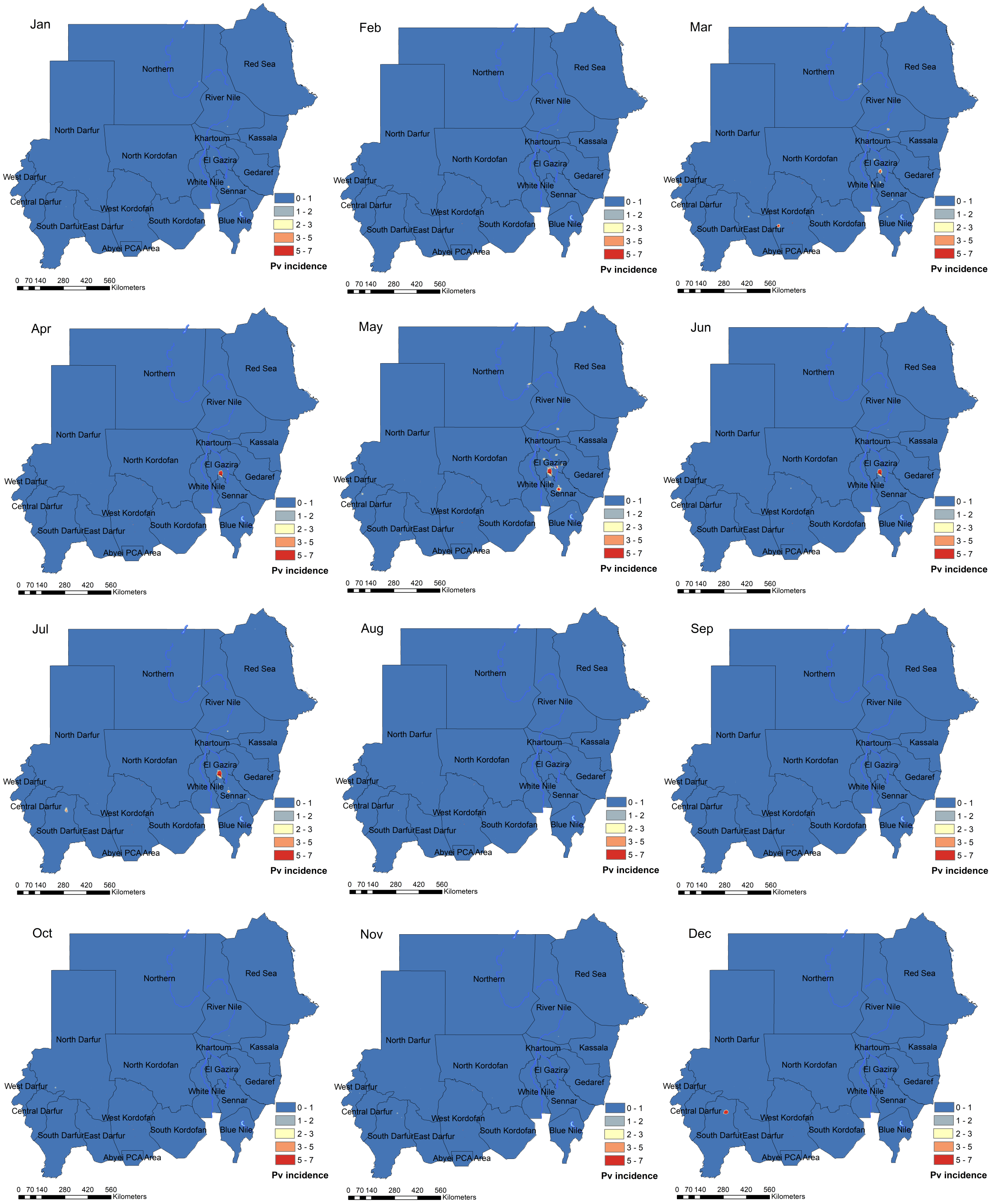

Figure 10. A fine-scale map (1 × 1 km) of the 25% credible interval of the incidence cases of *P. vivax* malaria per 1000 in each calendar month in Sudan inferred based on our spatiotemporal Bayesian model fit to the monthly routine surveillance data between 2017-2019. These figure were created in ArcGIS (`https://www.arcgis.com`) using the ArcMap package.

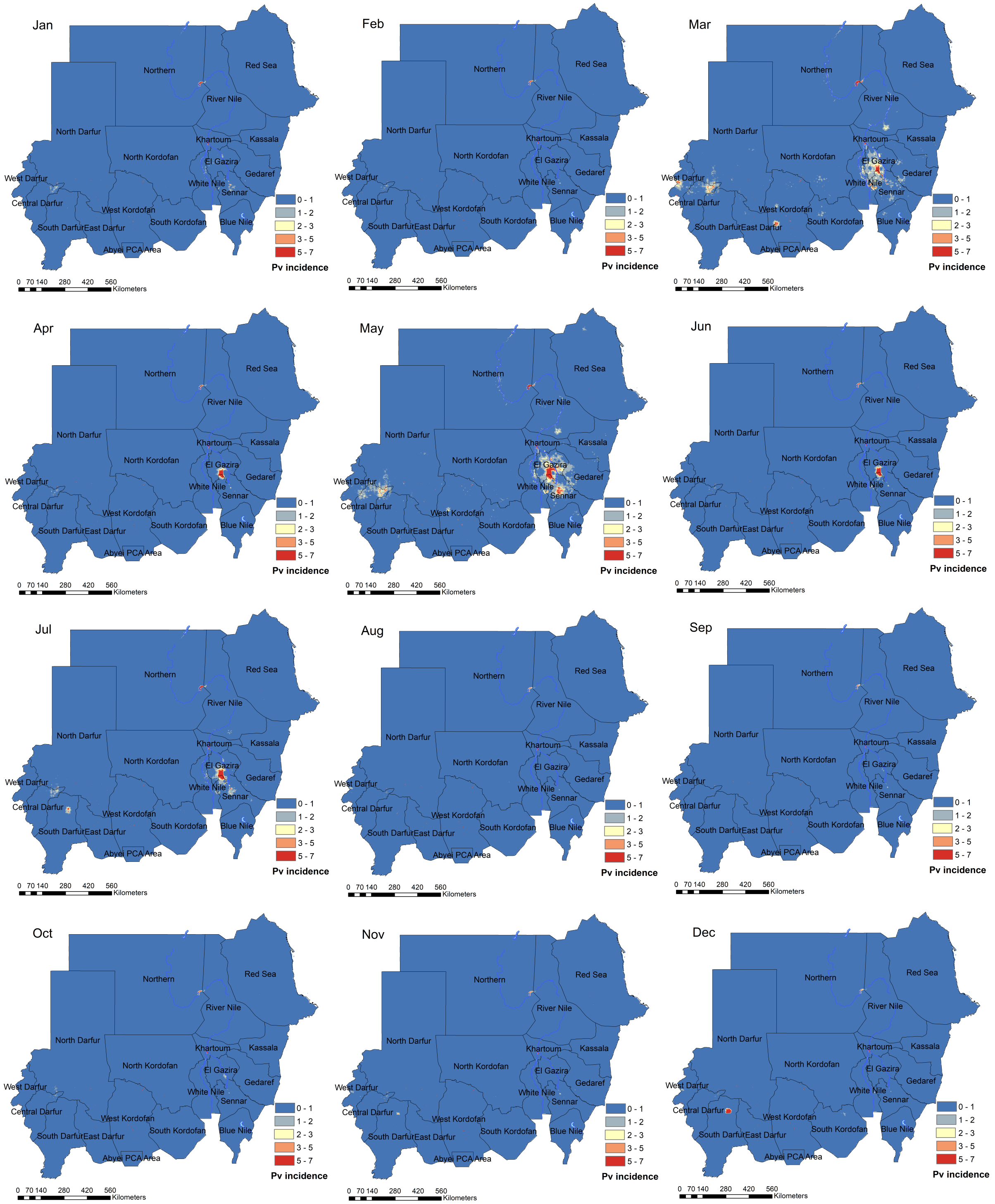

Figure 11. A fine-scale map (1 × 1 km) of the 68% credible interval of the incidence cases of *P. vivax* malaria per 1000 in each calendar month in Sudan inferred based on our spatiotemporal Bayesian model fit to the monthly routine surveillance data between 2017-2019. These figure were created in ArcGIS (`https://www.arcgis.com`) using the ArcMap package.

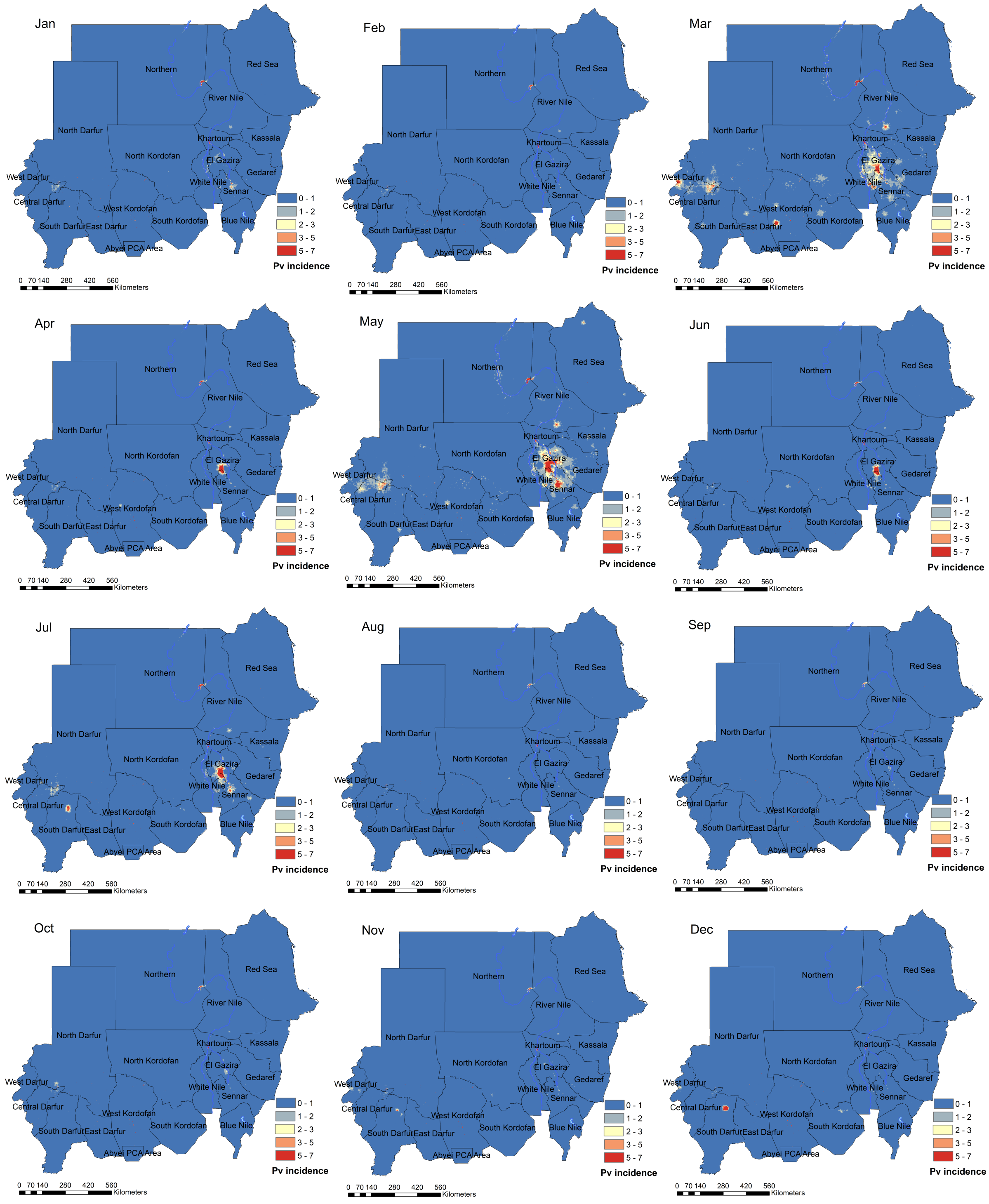

Figure 12. A fine-scale map (1 × 1 km) of the 95% credible interval of the incidence cases of *P. vivax* malaria per 1000 in each calendar month in Sudan inferred based on our spatiotemporal Bayesian model fit to the monthly routine surveillance data between 2017-2019. These figure were created in ArcGIS (`https://www.arcgis.com`) using the ArcMap package.
